# Gastrodin combined with donepezil alleviates cognitive dysfunction in Alzheimer’s disease mice by improving mitochondrial function through AMPK/PGC-1α/SIRT3 axis

**DOI:** 10.3389/fnagi.2026.1978729

**Published:** 2026-10-27

**Authors:** Junwen Wang, Jing Wu, Fenrong Liang, Shulei Huang, Longfei Liu, Ying Huang, Yiming Wang

**Affiliations:** 1 School of Clinical Medicine, Guizhou Medical University, Guiyang, Guizhou, China; 2 Department of Psychosomatic Medicine, The Second People’s Hospital of Guiyang, Guiyang, Guizhou, China; 3 Department of Neurology, The Second People’s Hospital of Guiyang, Guiyang, Guizhou, China; 4 Department of Psychiatry, Affiliated Hospital of Guizhou Medical University, Guiyang, Guizhou, China

**Keywords:** Alzheimer’s disease, AMPK/PGC-1α/SIRT3 axis, donepezil, gastrodin, mitochondrial function

## Abstract

**Objective:**

This study aims to explore whether gastrodin (Gas) combined with donepezil (Don) has a neuroprotective effect on Alzheimer’s disease (AD) mice by regulating the AMPK/PGC-1α/SIRT3 signaling axis.

**Methods:**

HT22 cells were transfected with SIRT3 siRNA or control siRNA. Cells were then treated with the AMPK inhibitor, Don, Gas, or their combination. Subsequently, HT22 cells were treated with Amyloid beta 1–42 (Aβ 1–42) to induce an *in vitro* AD model. Cell viability was detected by CCK8 assay. DCFH-DA fluorescent probe was used to evaluate cell reactive oxygen species (ROS). Adenosine triphosphate (ATP) content was detected by luciferase chemiluminescence method. Cell membrane potential was measured by flow cytometry. For *in vivo* experiments, WT mice and APP/PS1 mice were treated with Don, Gas or a combination of drugs. Morris water maze test, Y-maze test and novel object recognition test were used to evaluate the cognitive impairment of mice. The pathological changes of hippocampus were detected by HE staining, and the expression of Aβ protein in hippocampus and cortex was detected by IHC. In addition, the protein and mRNA expressions of APP, Tau, and AMPK/PGC-1α/SIRT3 signaling axis were determined by Western blot and RT-QPCR.

**Results:**

Gas combined with Don significantly improved cognitive dysfunction, reduced Aβ plaque deposition in the cortex and hippocampus, up-regulated AMPK/PGC-1α/SIRT3 axis, and inhibited the abnormal phosphorylation of Tau and APP in APP/PS1 mice compared with the model group and the Don group (*p* < 0.05). *In vitro* cell experiments confirmed that the combination of the two drugs could improve cell viability, inhibit cell apoptosis, restore ATP level, reduce ROS accumulation, stabilize mitochondrial membrane potential, and improve mitochondrial function (*p* < 0.05). Molecular experiments and complementary experiments showed that Gas combined with Don activated AMPK/PGC-1α/SIRT3 signaling axis (*p* < 0.05). Silencing SIRT3 or inhibiting AMPK could reverse the protective effect of the combined regimen (*p* < 0.05).

**Conclusion:**

Gas combined with Don can maintain mitochondrial homeostasis through AMPK/PGC-1α/SIRT3 pathway, alleviate AD-relaated pathological damage, and exert neuroprotection.

## 1 Introduction

Alzheimer’s disease (AD), a neurodegenerative disorder characterized by progressive cognitive impairment and memory loss, is the most common cause of dementia affecting approximately 40 million people worldwide and has become a major public health problem that seriously threatens the health of the elderly (Detka et al., 2024). The typical pathological features of AD include senile plaques formed by extracellular amyloid-β (Aβ) deposition and neurofibrillary tangles formed by intracellular hyperphosphorylated tau protein aggregation (Courade et al., 2025). At present, the etiology of AD is still unclear, and there are no effective measures for clinical prevention and treatment (Nowell et al., 2023). Mitochondrial dysfunction plays a key role in the pathogenesis of AD (D'Alessandro et al., 2025). As the “energy factory” of cells, mitochondria are not only responsible for Adenosine triphosphate (ATP) production, but also participate in calcium homeostasis regulation and oxidative stress management (D'Alessandro et al., 2025). In AD brain, impaired mitochondrial function is manifested as decreased mitochondrial membrane potential, decreased ATP production, excessive production of reactive oxygen species (ROS), and imbalance of mitochondrial dynamics (Lei et al., 2026). More importantly, Aβ can accumulate in mitochondria and directly induce the opening of mitochondrial permeability transition (MPT), which further aggravates mitochondrial damage (Kimura et al., 2021). Therefore, improving mitochondrial function has become one of the important directions for AD drug development.

The AMPK/PGC-1α/SIRT3 signaling axis is one of the core pathways regulating mitochondrial function (Wang et al., 2025). AMP-activated protein kinase (AMPK) is a cellular energy sensor that is activated in response to energy stress. Furthermore, it phosphorylates and activates the downstream peroxisome proliferator-activated receptor gamma coactivator-1α (PGC-1α) (Wang et al., 2025). PGC-1α is a master regulator of mitochondrial biosynthesis and is able to coordinate the expression of nuclear encoded mitochondrial genes (Ren et al., 2024). As the most important deacetylase in mitochondria, SIRT3 can be up-regulated by PGC-1α, and regulate key processes such as oxidative phosphorylation, antioxidant defense and mitochondrial dynamics through deacetylation of mitochondrial proteins (Wang et al., 2022). Recent studies have found that activation of AMPK/PGC-1α/SIRT3 pathway can significantly improve mitochondrial function, reduce oxidative stress, and play a neuroprotective role in various disease models such as AD and ischemia–reperfusion (Wang et al., 2025; Gu et al., 2023; Zhu et al., 2024). Gastrodin (Gas), the main active phenolic side component of *Gastrodia elata Bl.*, has significant neuroprotective and mitochondrial regulatory effects. Gas can improve neuronal mitochondrial and synaptic damage by up-regulating SIRT3 expression, inhibiting oxidative stress and neuroinflammation, reducing Aβ deposition and Tau hyperphosphorylation (Lei et al., 2026; Zhu et al., 2024). In addition, the first-line clinical drug donepezil (Don) can not only inhibit cholinesterase classically, but also promote mitochondrial biosynthesis and improve mitochondrial functional stability by activating AMPK/PGC-1α pathway to exert anti-AD effects (Kim et al., 2016; Angelova et al., 2024). However, Don still has some limitations, including that it can only improve cognitive symptoms and has obvious side effects.

Current treatments for AD, which target a single pathological mechanism, provide only temporary relief and rarely alter disease progression. Multi-target drugs (MTDs) can target multiple subpathologies, including impaired pathological protein aggregation, and their potential in improving the treatment of AD has attracted much attention, especially the combination strategy of western drugs and natural drugs (Angelova et al., 2024). Existing studies mostly focus on the independent mechanisms of Gas or Don in the intervention of AD, and there are obvious complementary pathways in the regulatory characteristics of Gas and Don targeting mitochondria. However, there is no systematic study to elucidate the mitochondrial mechanism of Gas combined with Don in anti-AD. Whether the combination of Gas and Don can improve mitochondrial dysfunction and reverse cognitive impairment more effectively than monotherapy through the AMPK/PGC-1α/SIRT3 signaling pathway remains to be determined. Therefore, taking mitochondrial dysfunction as the breakthrough point, this study explored the neuroprotective effects of Gas combined with Don and its molecular mechanism in AD cell and animal models, aiming to provide a new experimental basis and theoretical support for the multi-target drug combination strategy of AD.

## 2 Materials and methods

### 2.1 Animal experiments and experimental design

Eight 6-month-old C57BL/6J wild-type male mice and 32 male APP/PS1 double transgenic mice were obtained from Hangzhou Ziyuan Laboratory Animal Technology Co., Ltd. (license number: SCXK (Zhe) 2024–0004). After 7 days of adaptive feeding, C57BL/6J wild type male mice were used as control group, that is, wild-type WT group. APP/PS1 mice were randomly divided into APP/PS1 group, APP/PS1 + Don group, APP/PS1 + Gas group and APP/PS1 + Don+Gas group, with 8 mice in each group. All drugs were prepared with normal saline. Don (2 mg/kg/d) (HY-14566, MCE) and Gas (50 mg/kg/d) (HY-N0115, MCE) were administered by intragastric administration and intraperitoneal injection, respectively. Gas was administered 0.5 h after Don administration, once daily for 8 weeks. The control and model groups were given the same volume of solvent. All animals were bred and housed under a constant 22 °C-24 °C, 50 to 60% humidity, 12/12 h light–dark cycle, and had AD libitum access to food and water. Animal experiments have been approved by the Animal Ethics Committee of Guizhou Medical University (Approval No. 2602181).

### 2.2 Morris water maze (MWM) test

This test is used to assess memory function in mice (Sun et al., 2021). This was performed 1 week before the end of the dosing period. Five days of directional navigation experiment (escape latency and swimming path were recorded) and 6 days of space exploration experiment (residence time of target quadrant and number of crossings the original platform were recorded) were included. Data were recorded and analyzed using a video analysis system.

### 2.3 Y-maze test

The experiment was designed to evaluate the memory and exploration abilities of mice (Wang et al., 2023). The Y-shaped maze consisted of three arms connected in the middle into a “Y” shape with a 120° Angle between the arms. A video camera and software were used for recording during the test. During the acquisition phase, the new arm was blocked to enable the mice to explore the other two arms for 10 min. During the test phase, all three arms were opened. The mice were allowed to explore the maze freely for 8 min, and the spontaneous alternating response rate or the total number of entries into each arm was recorded.

### 2.4 New object recognition test

Novel object recognition experiment: used to evaluate the improvement of recognition memory ability (Zhang Y. et al., 2025). The experiment lasted for 3 days. The adaptation period (day 1): Mice were placed individually in the empty experiment box and explored freely for 10 min. The experiment was adapted once a day for 2 consecutive days to reduce the interference of novel environment on the experimental results. Familiarization period (morning of day 3): Two identical objects (objects A1 and A2) were placed symmetrically on both sides of the box wall in the experimental box. Mice were placed into the box facing the wall and explored freely for 10 min. The time of active exploration of each object was recorded (exploration was defined as the distance between the nose tip of the mouse and the object ≤ 2 cm or the active smell or touch of the object). Test period (afternoon of day 3): One of the familiar objects (such as A2) was replaced with a new object B (similar in shape and size to A2 but different). Mice were placed in the box and explored freely for 10 min. The time spent exploring the familiar object A1 and the novel object B was recorded.

### 2.5 Brain collection and brain tissue preparation

After the behavioral experiments, the mice were anesthetized by intraperitoneal injection of pentobarbital sodium, and then underwent cardiac perfusion. The hippocampus and cortical regions of the brain were collected and preserved in formaldehyde for embedding, subsequent sectioning, and staining. Some mice were sacrificed after anesthesia, and the hippocampus was quickly collected at low temperature for other experimental detections.

### 2.6 Hematoxylin and eosin (HE) staining

The cut brain tissue sections were deparaffinized and rehydrated stained with hematoxylin and eosin (Biyun Tian, CO105S) then dehydrated in graded ethanol, cleared in xylene, and mounted with neutral gum. Three random fields of view at 100x and 400x were selected under the microscope to observe the tissue morphology.

### 2.7 Immunohistochemistry

The sections were sequentially dehydrated with xylene, 100%, 95 and 80% ethanol, then rinsed with tap water, and subsequently subjected to high-pressure antigen retrieval. Two liters of citrate buffer solution was prepared as the antigen retrieval solution. The sections were placed in a pressure cooker and boiled to undergo thermal retrieval. After marking the tissue regions with an immunohistochemical pen, they were placed in an incubation box, and 3% H₂O₂ was added for incubation. After washing with Phosphate Buffered Saline (PBS), the sections were air-dried and then added with primary antibody Aβ (ab201060, abcom) for incubation at 37 °C. Subsequently, the secondary antibody was used, and PBS was washed before adding DAB chromogenic agent (DA1010, Solarbio). The staining process was observed under a microscope when positive staining appeared, and the reaction was terminated. The sections were re-stained with hematoxylin, then differentiated with hydrochloric acid alcohol, re-blotched with lithium carbonate, dehydrated, cleared, and mounted. Three random fields of view at 200x and 400x were selected under the microscope to observe the tissue morphology.

### 2.8 Drug preparation

The Aβ1-42 peptide was prepared according to a previously published protocol (Sanjay et al., 2024). As follows, Aβ1-42 (107761–42-2, MKbio) was pre-treated with hexafluoroisopropanol (HFIP). In brief, 1 mg of Aβ1-42 was suspended in 1 mL of HFIP and equally divided into different Eppendorf tubes (100 μL each). The tubes were kept open and placed in a covered container at room temperature for 16–18 h to evaporate all HFIP. After complete evaporation of HFIP, the equally divided samples were stored at −80 °C. Before treating the cells, Aβ1-42 was mixed with 5.5 μL DMSO (ID9011, Solarbio) and 270 μL PBS (P1020, Solarbio) to obtain the final concentration of Aβ1-42. The Aβ1-42 was incubated at 4 °C for 24 h before use and further diluted with DMEM medium (PM150210, Procell) for different treatments. Tubocurarine, Don, and AMPK inhibitor Compound C (HY-13418, MCE) were dissolved in DMSO to form stock solutions.

### 2.9 Cell experiment grouping and drug intervention

Mouse hippocampal neuronal cells HT-22 were purchased from Wuhan Promesa Life Science Technology Co., Ltd. They were seeded in dulbecco’s modified eagle medium (DMEM) containing 10% Foetal Bovine Serum (FBS) (AUS-01S-02, Cell box) + 1% Penicillin–Streptomycin Solution (P/S) (P1400, Solarbio) and incubated in a 37 °C, 5% CO2 incubator. The cell experiments were divided into Control group, Model group, Don group, Gas group, and Don+Gas group. After 24 h of cell seeding, the medium was replaced. The Model group was treated with 10 μM Aβ1-42. The Don group was treated with 10 μM Aβ1-42 and 50 μM Don, the Gas group was treated with 10 μM Aβ1-42 and 20 nM Gas, and the Don+Gas group was treated with the combination of the model group and Don. Various detection experiments were conducted 24 h after the treatment.

### 2.10 Transfection of siRNA and intervention with AMPK inhibitors

The experiments were divided into Control, Model, Don+Gas+siRNA NC, Don+Gas+si-SIRT3, si-SIRT3 and Don+Gas+AMPK inhibitor. Before the model induction, si-SIRT3 and sirNA-NC were transfected into the cells of the transfection group using siRNA transfection reagent (L3110, Solarbio) 24 h after the cells were inoculated to knock down the expression of SIRT3 in the cells. The most effective siRNA: sense: 5’-UCGCCUCAAAGCUG GUUGA-3′, antisense: 5 ‘-UCAACCAGCUUUGAGGCGA-3′. After 6 h, the medium was replaced with complete medium, and SIRT3 mRNA and protein expression levels were measured after continued culture for 24 h. The AMPK inhibitor group was pretreated with 10 μM Compound C for 2 h after transfection. Subsequently, the model induction and combined administration of Don+Gas were carried out in the above way, and the Control group was given the corresponding solvent.

### 2.11 CCK8 assay for cell viability

Cells were seeded into 96-well plates at 5 × 10^3^ cells per well at 100 μL/well. The intervention methods described above were applied. After 24 h of treatment, 10 μL of CCK8 reagent was added to each well, and the plates were incubated at 37 °C for 2 h. The optical density (OD) at 450 nm was measured using amicroplate reader.

### 2.12 Flow cytometry for mitochondrial membrane potential

Cells were inoculated at 5× 10^5^ per well into a 6-well plate and allowed to attach in the incubator. The next day, the culture medium was discarded, and the intervention methods described above were applied. After 24 h of treatment cells were collected and resuspended to prepare a single-cell suspension. Cells were added with 0.5 mL of culture medium and 0.5 mL of freshly prepared TMRE staining working solution (CA3660, Solarbio), mixed, and incubated at 37 °C for 20 min. After incubation, the supernatant was aspirated: cells were washed twice with freshly prepared TMRE staining buffer (1X) and resuspended in an appropriate volume of TMRE staining buffer (1X). Cells were then analyzed by flow cytometry.

### 2.13 Flow cytometry for cell apoptosis

Cells were inoculated at 1×10^6^ per well into a 6-well plate and allowed to adhere for 24 h. The intervention methods described above were applied. After 24 h of treatment, cells were collected and washed. Cells were stained with the AnnexinV-APC/PI kit (CA3580, Solarbio). The cell pellet was resuspended in 600 μL of binding solution to obtain asingle-cell suspension. Then, 100 μL of the suspension was transferred to an EP tube, and 5 μL of APC staining solution and 5 μL of PI staining solution were added. After incubation at room temperature in the dark for 15 min, 400 μL of binding solution was added, and cell apoptosis was detected by flow cytometry.

### 2.14 Detection of adenosine triphosphate (ATP) content in cells

The detection of intracellular ATP levels was carried out strictly in accordance with the manufacturer’s instructions of the ATP content detection kit (BC0305, Solarbio). The absorbance value was read at a wavelength of 340 nm on a microplate reader.

### 2.15 Detection of reactive oxygen species (ROS) levels in cells

The detection of intracellular ROS levels was also conducted strictly in accordance with the manufacturer’s instructions of the ROS content detection kit (CA1420, Solarbio). The fluorescence intensity of the samples was detected using a multi-functional fluorescence microplate reader. The excitation wavelength was 488 nm and the emission wavelength was 525 nm. The fluorescence readings of each group were recorded.

### 2.16 RT-QPCR

Firstly, the APP/PS1 mouse hippocampal tissue was ground and homogenized in a buffer. Then, total RNA was extracted and reverse transcribed into complementary DNA (cDNA) using the reverse transcription kit (RR047A, TaKaRa). Subsequently, the mRNA levels were quantified using the SYBR QPCR mixture (E096-01B, Novoprotein). The relative mRNA expression of AMPK, PGC-1α, SIRT3 and Tau was calculated using the 2 − ΔΔCt method, with GAPDH as the internal reference. (The QPCR primers are detailed in Table 1).

**Table 1:** Forward and reverse primer sequences for RT-QPCR.

Genes	Primer sequence
SIRT3	Forward: 5’-GAGCGGCCTCTACAGCAAC-3’
	Reverse: 5’-GGAAGTAGTGAGTGACATTGGG-3’
AMPK	Forward: 5’-TTCGGGAAAGTGAAGGTGGG-3’
	Reverse: 5’-TCTTCTGCCGGTTGAGTATCT-3’
PGC-1α	Forward: 5’-TATGGAGTGACATAGAGTGTGCT-3’
	Reverse: 5’-CCACTTCAATCCACCCAGAAAG-3’
Tau	Forward: 5’-CGCTGGGCATGTGACTCAA-3’
	Reverse: 5’-TTTCTTCTCGTCATTTCCTGTCC-3’
GAPDH	Forward: 5’-TGGCCTTCCGTGTTCCTAC-3’
	Reverse: 5’-GAGTTGCTGTTGAAGTCGCA-3’

### 2.17 Western blot analysis

Cells and hippocampal tissue homogenates were collected and lysed with lysis buffer to ensure completelysis. Then, centrifugation was performed at 12,000 g for 15 min at 4 °C, and the supernatant was collected. After protein electrophoresis and membrane transfer, the PVDF membrane were blocked with skim milk. Subsequently, the PVDF membrane was placed into the primary antibody solution (p-AMPK (AF3423, Affinity), AMPK (AF6423, Affinity), PGC-1α (AF5135, Affinity), and P-AMPK (AF6423, Affinity), SIRT3 (AF5135, Affinity), p-APP (AF30844, Affinity), APP (AF6084, Affinity), p-Tau (AF3148, Affinity), Tau (AF6141, Affinity) and β-actin (bsm-63325R, Bioss)) in and incubated overnight at 4 °C. After incubation with primary antibodies, the cells were washed with TBST. This antibody was incubated with the corresponding secondary antibody (SA00001-2, Proteintech) for 1 h at room temperature. Subsequently, after washing the PVDF membrane in TBST, the developer solution was uniformly added and imaged by a gel imager. Protein band intensities were analyzed using ImageJ software.

### 2.18 Statistical analysis

All statistical analyses were performed using GraphPad Prism 10.1.2. Data were presented as mean ± standard error of the mean (SEM), and a *p* < 0.05 was considered statistically significant. Normality of data distribution was assessed by the Shapiro–Wilk test, and homogeneity of variances was verified using Levene’s test. For data conforming to both normality and homogeneity of variances, one-way analysis of variance (ANOVA) was applied for intergroup comparisons. If the normality assumption was violated, nonparametric tests were employed: the Mann–Whitney test for comparisons between two groups and the Kruskal–Wallis test for comparisons among more than two groups.

## 3 Results

### 3.1 Gas combined with Don can alleviate cognitive dysfunction in AD model mice

We used MWM and Y-maze tests as well as novel object recognition tests to evaluate the effects of Don combined with Gas on cognitive dysfunction in AD mice after 56 days of administration. The results of MWM (Figures 1A,B) showed that compared with the WT group, the APP/PS1 model mice had significantly reduced the number of platform crossings (*p* < 0.05) and the time spent in the target quadrant (*p* < 0.001), suggesting that the model mice had spatial learning and memory impairment. The number of platform crossings was increased with Don or Gas alone or in combination, although the differences did not reach statistical significance. In the statistical quadrant residence time, the administration of Don or Gas alone partially improved the cognitive deficits (*p* < 0.05), and Gas and Don treatment further restored the spatial memory ability of APP/PS1 mice (*p* < 0.01). The results of Y maze test (Figure 1C) showed that the spontaneous altercountry rate of APP/PS1 mice was significantly decreased (*p* < 0.001), reflecting the impairment of working memory. Gas combined with Don could significantly improve the spontaneous altercountry behavior of mice (*p* < 0.01), and the effect was better than that of GAS or DON alone. In the novel object recognition test (Figure 1D), the novel object recognition index of the APP/PS1 group was significantly decreased (*p* < 0.001), and the recognition ability of the mice was significantly improved after the combination of Don and Gas treatment (*p* < 0.01). Figures 1E–G shows the trajectory heatmaps of each group of mice for the MWM, Y-maze test, and novel object recognition test, respectively. These results indicated that Gas combined with Don exerted enhanced protective effects in alleviated cognitive dysfunction in APP/PS1 transgenic mice.

**Figure 1:**
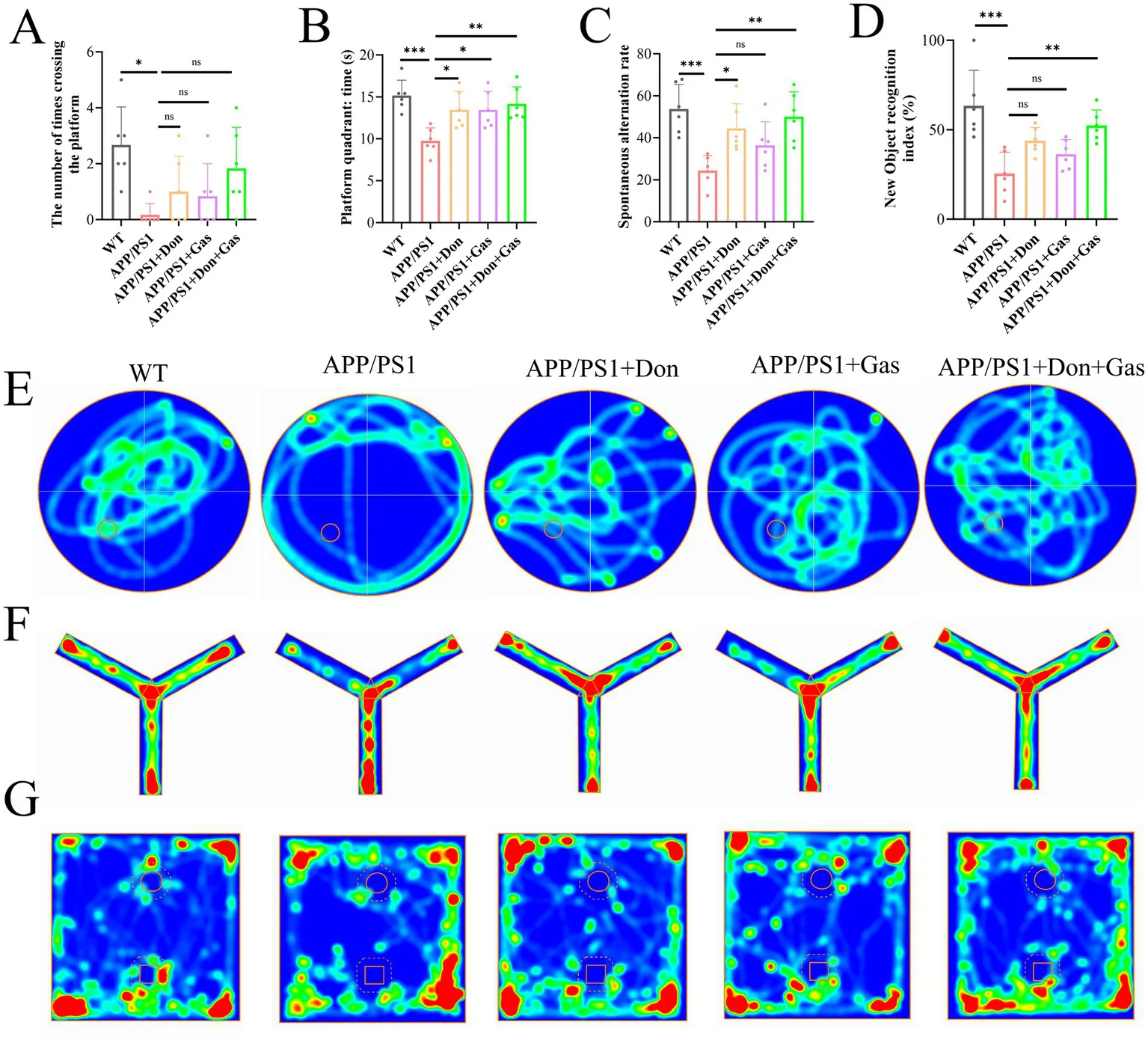
Behavioral tests evaluate the cognitive dysfunction in AD model mice. **(A,B)** MWM test (*n* = 6): **(A)** Number of platform crossings; **(B)** Time spent in target quadrant; **(E)** Representative motion trajectories of mice. **(C)** Y-maze test (*n* = 6): Spontaneous alternation rate. **(D)** Novel object recognition test (*n* = 6): Novel object recognition index. **(E–G)** Representative trajectory heat maps of the MWM **(E)**, Y-maze **(F)**, and novel object recognition **(G)** tests. Data are presented as mean ± SEM. *p < 0.05, **p < 0.01, ***p < 0.001, ^ns^*p* > 0.05.

### 3.2 Gas combined with Don alleviated the pathological features of AD model mice

AD is closely related to the damage of hippocampal neurons (Figure 2A). The HE staining analysis showed (Figure 2B) that the neuronal morphology was normal in the wild-type (WT) group, while the neurons in the APP/PS1 group showed signs of atrophy, disordered arrangement, excessive staining, and cell body deformation. The cell distribution in the Don group, Gas group, and the combined intervention group was more uniform compared to that of AD mice, indicating significant recovery of neural damage, and the combined intervention of Gas and Don showed the most significant improvement. Aβ is a key pathological factor of AD and promotes brain neuroinflammation (Kim et al., 2021). Therefore, we evaluated it. The immunohistochemical results showed (Figures 2C–F) that the deposition of Aβ plaques in the cortex and CA area of the hippocampus of APP/PS1 mice was significantly increased compared to the WT mice (*p* < 0.0001). Don or Gas monotherapy could partially inhibit the aggregation of Aβ in the brain (*p* < 0.05); the combined intervention of the two significantly reduced the proportion of Aβ positive area in the cortex and hippocampus (*p* < 0.0001), exhibiting a greater inhibitory effect on Aβ deposition than either monotherapy. These results suggest that, compared with either single intervention, the combination of Gas and Don more effectively alleviated Aβ pathological deposition and neuronal damage in APP/PS1 mice.

**Figure 2:**
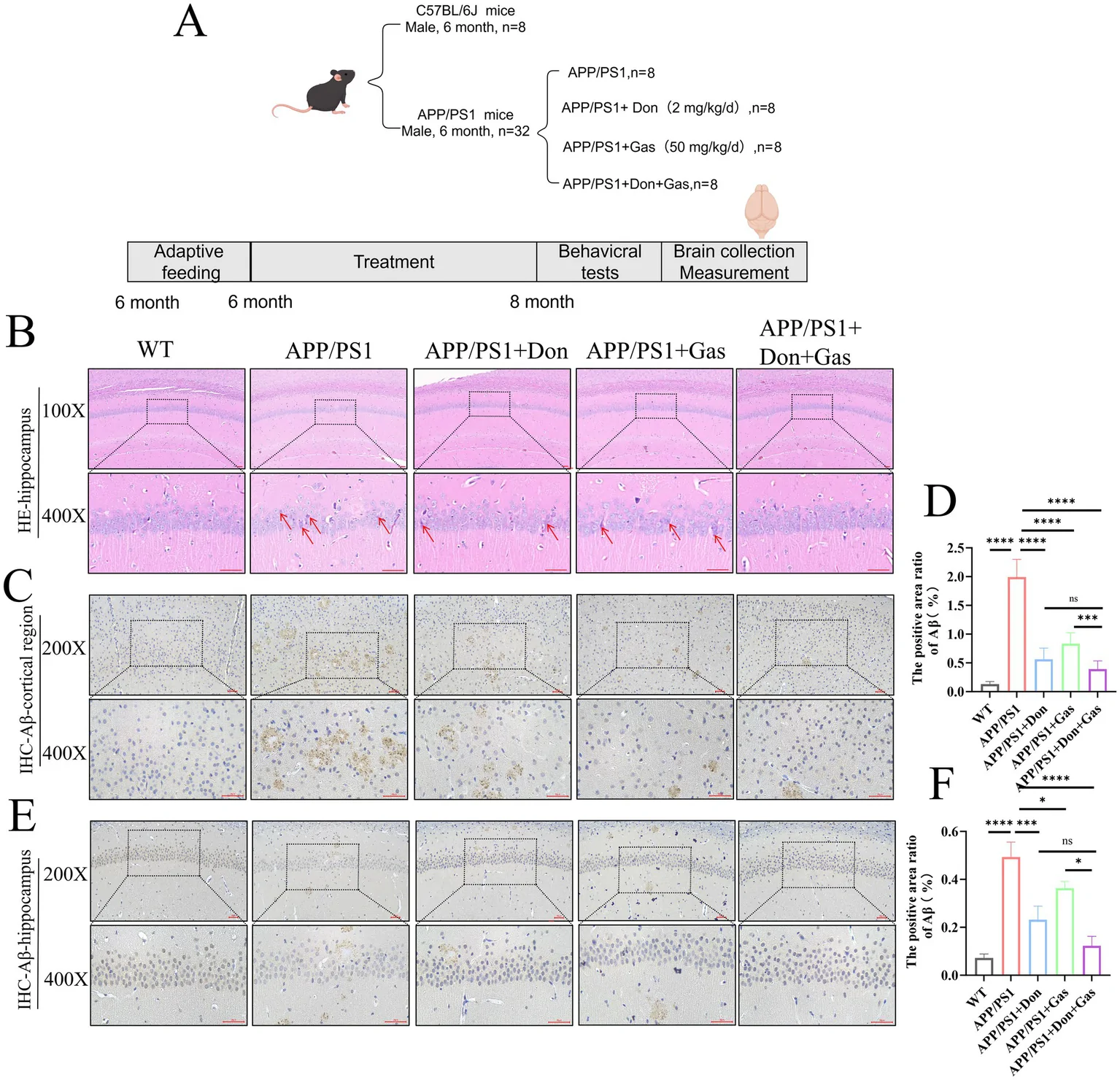
HE and immunohistochemical assays for evaluating neuronal damage in AD mice. **(A)** Schematic diagram of the animal experimental schedule (Jiang et al., 2025). **(B)** HE staining of hippocampal tissues in each group (*n* = 3). **(C,D)** Immunohistochemical detection of Aβ expression in the cortical tissues of each group of mice (*n* = 6), magnification 100x, 400x. **(E,F)** Immunohistochemical detection of Aβ expression in the hippocampal tissues of each group of mice (*n* = 6), magnification 100x, 400x. Scale bar: 50 μM. Data are presented as mean ± SEM. *p < 0.05, **p < 0.01, ***p < 0.001, ****p < 0.0001, ^ns^*p* > 0.05.

### 3.3 Gas combined with Don alleviates cognitive dysfunction in AD mice through the AMPK/PGC-1α/SIRT3 axis

The QPCR results (Figures 3A–D) showed that compared with the WT group, the transcriptional levels of Ampk, Pgc-1α, and Sirt3 genes in the brain tissues of APP/PS1 model mice were significantly downregulated, while the expression of Tau gene was significantly increased (*p* < 0.001). The single intervention of Don or Gas could partially upregulate the mRNA expression of AMPK, PGC-1α, and SIRT3, and reduce the Tau transcription level (*p* < 0.05); while the combined administration of Gas and Don could further significantly activate this pathway (*p* < 0.01), and the therapeutic effect was better than that of the single drug treatment group.

**Figure 3:**
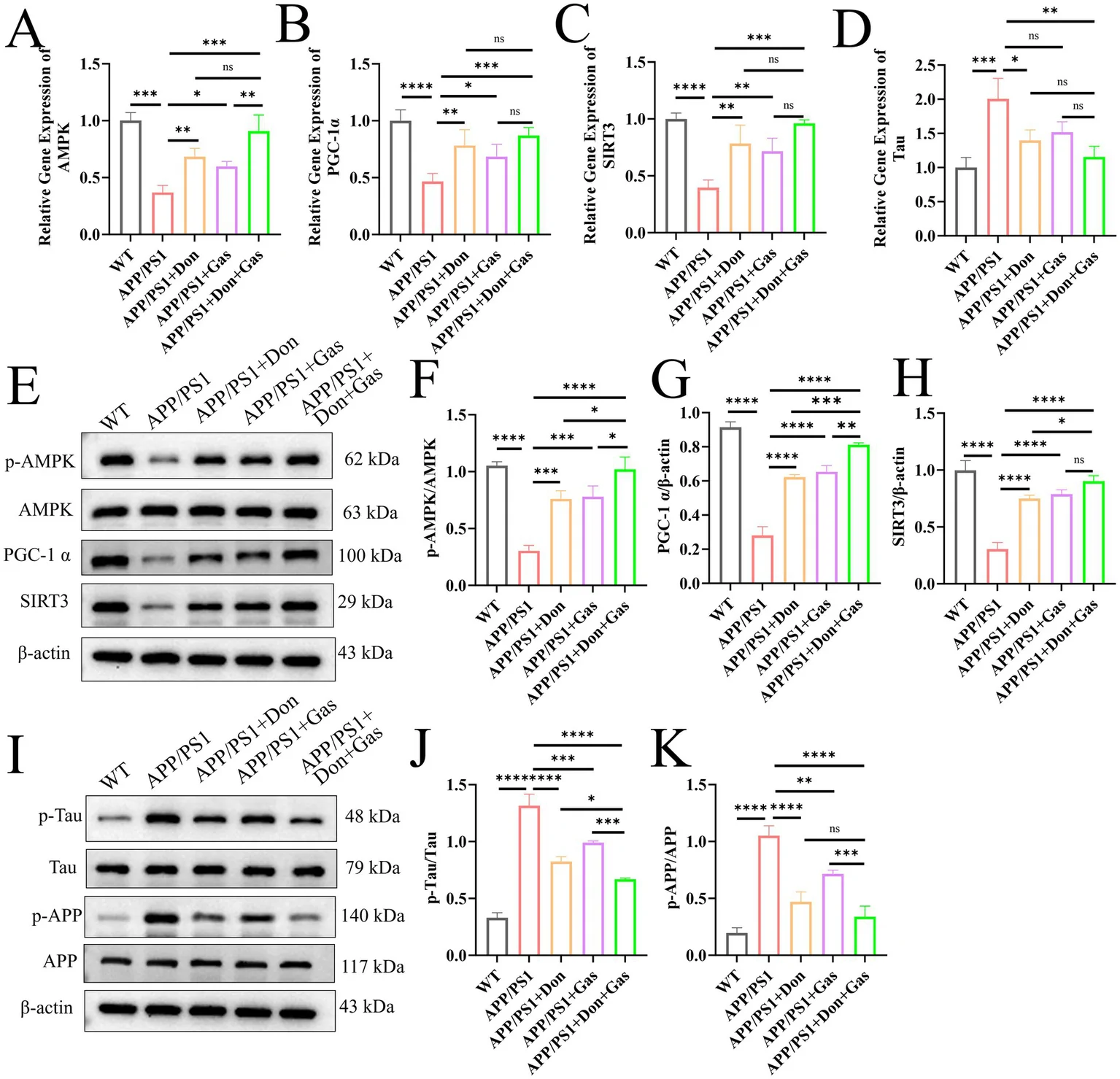
RT-QPCR and Western blot were used to detect the expression of the AMPK/PGC-1α/SIRT3 signaling axis, Tau, and APP in the hippocampal tissues of each group of mice. **(A–D)** QPCR was used to measure the mRNA expression levels of AMPK, PGC-1α, SIRT3, and Tau in the hippocampal tissues (*n* = 3). **(E–K)** Western blot was used to detect the expression of the AMPK/PGC-1α/SIRT3 signaling axis, Tau, APP, and phosphorylated proteins in the hippocampal tissues of each group of mice (*n* = 3). Data are presented as mean ± SEM. *p < 0.05, **p < 0.01, ***p < 0.001, ****p < 0.0001, ^ns^*p* > 0.05.

The Western blot results further verified (Figures 3E–H) that compared with the WT group, the relative expression levels of p-AMPK/AMPK, PGC-1α, and SIRT3 proteins in the brain tissues of APP/PS1 mice were significantly decreased (*p* < 0.0001); after single intervention with Don or Gas, the expression of pathway proteins recovered (*p* < 0.0001), and the combined use of Don and Gas further up-regulated the protein levels of p-AMPK/AMPK, PGC-1α, and SIRT3.

In terms of the characteristic pathological proteins of AD, the ratios of p-Tau/Tau and p-APP/APP in the APP/PS1 model group were significantly increased (*p* < 0.0001), suggesting excessive Tau phosphorylation and abnormal activation of APP; Don or Gas alone slightly inhibited the accumulation of p-Tau and p-APP (*p* < 0.01), and the combined intervention could significantly reduce the ratios of p-Tau/Tau and p-APP/APP proteins (*p* < 0.0001), alleviating Tau phosphorylation and abnormal APP cleavage (Figures 3I–K).

The above results indicate that Gas and Don can effectively activate the AMPK/PGC-1α/SIRT3 signaling axis, inhibit excessive APP activation and Tau protein phosphorylation, and thereby improve the pathological damage related to APP/PS1 mice with AD.

### 3.4 Don combined with Gas improves mitochondrial energy metabolism and oxidative stress in the AD cell model

By inducing HT22 cell damage with Aβ42. As shown in Figure 4, compared with the control group, the cell viability in the model group significantly decreased. When Don, Gas, or the combined intervention of Don and Gas were administered alone, they all significantly increased the cell viability of HT22 cells (*p* < 0.0001). At the same time, compared with the control group, the ATP and mitochondrial membrane potential levels of the model group cells significantly decreased (*p* < 0.0001), and the ROS level significantly increased (*p* < 0.0001); compared with the model group, the individual interventions of Don, Gas, and the combined intervention could all significantly reverse these phenomena, with the combined intervention of Don and Gas showing a more significant effect (*p* < 0.001). Therefore, Gas and Don have a more protective effect on AD model cells and promote their survival.

**Figure 4:**
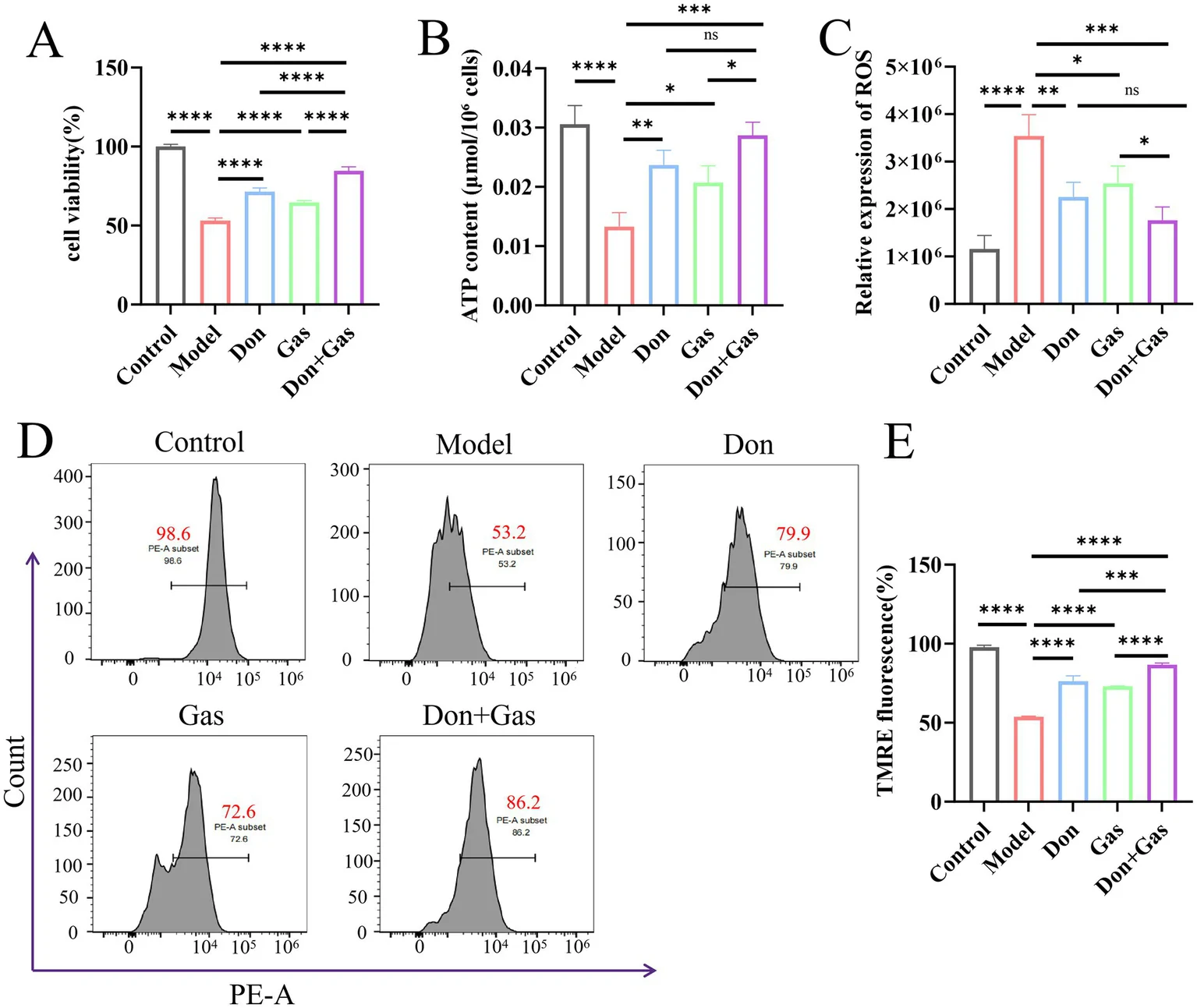
Evaluation of cell viability, mitochondrial energy metabolism and oxidative stress levels in each group. **(A)** Cell viability detected by CCK8 assay (*n* = 3). **(B)** Cellular ATP content (*n* = 3). **(C)** Relative ROS expression level (*n* = 3). **(D,E)** Flow cytometry analysis of mitochondrial membrane potential (TMRE staining) and quantitative statistics (*n* = 3). Data are presented as mean ± SEM. *p < 0.05, **p < 0.01, ***p < 0.001, ****p < 0.0001, ^ns^*p* > 0.05.

### 3.5 Don combined with Gas improves the AD cell model through AMPK/PGC-1α/SIRT3 pathway

The results of QPCR and Western blot showed that compared with the control group, the AMPK/PGC-1α/SIRT3 pathway in the Model group was significantly inhibited, accompanied by an increase in p-Tau and p-APP protein levels (*p* < 0.05). The combined intervention of Don and Gas could up-regulate the gene and protein expressions of the molecules in the AMPK/PGC-1α/SIRT3 pathway, and significantly reduce the ratio of p-Tau/Tau and p-APP/APP (*p* < 0.05). This result further indicates that Gas combined with Don activates the AMPK/PGC-1α/SIRT3 pathway, inhibits excessive phosphorylation of Tau and APP, and thereby exerts neuroprotective effects (see Figure 5).

**Figure 5:**
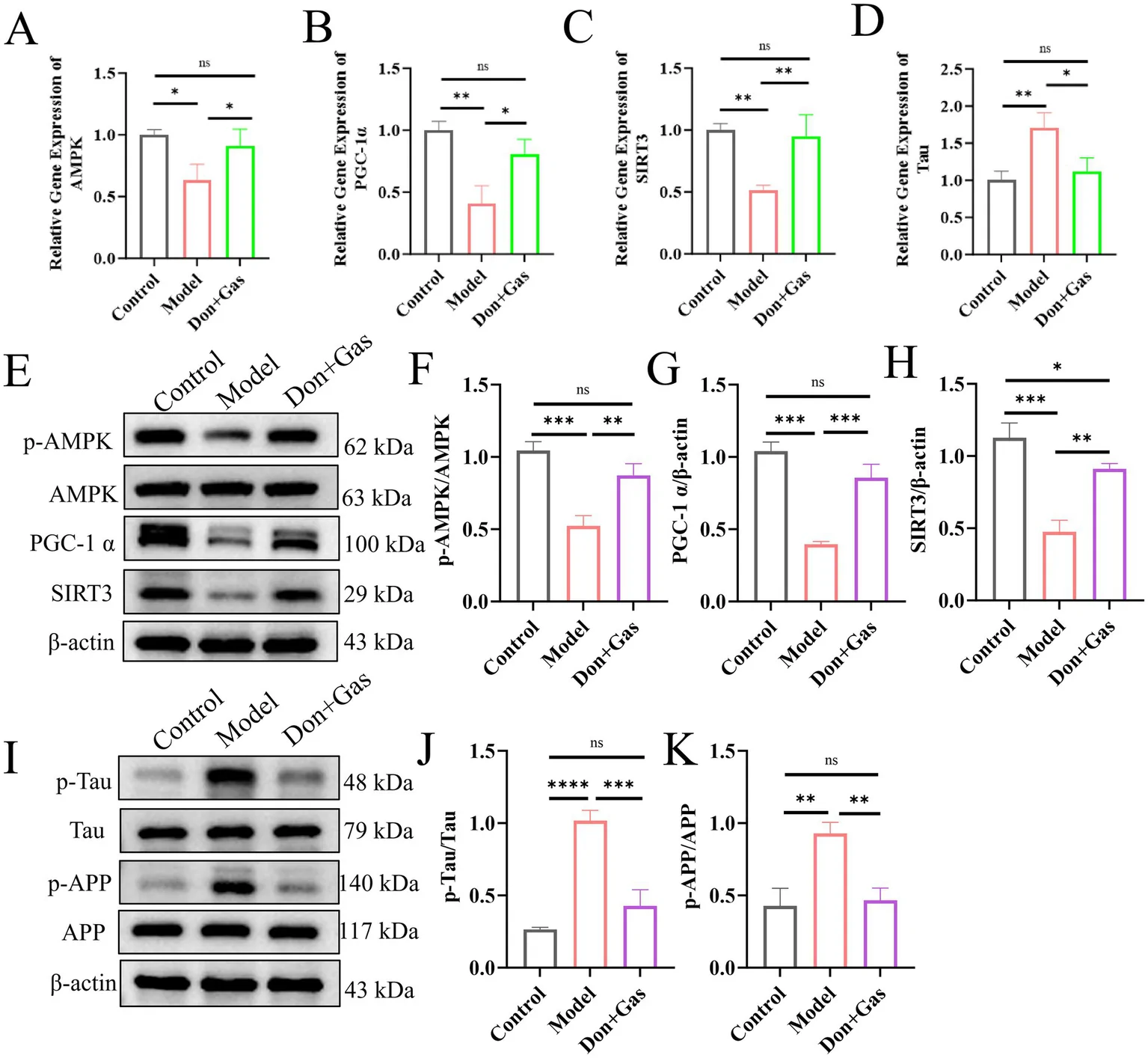
RT-QPCR and Western blot were used to detect the expression levels of the AMPK/PGC-1α/SIRT3 axis, Tau, and APP in each group of cells. **(A–D)** Relative mRNA expression of Ampk, Pgc-1α, Sirt3, and Tau was measured by RT-QPCR (*n* = 3). **(E–H)** Western blot was used to detect the protein expressions of p-AMPK, AMPK, PGC-1α, and SIRT3 in the hippocampal tissues of each group of mice (*n* = 3). **(I–K)** Western blot was used to detect the protein expressions of p-Tau, Tau, p-APP, and APP in the hippocampal tissues of each group of mice (*n* = 3). Data are presented as mean ± SEM. *p < 0.05, **p < 0.01, ***p < 0.001, ****p < 0.0001, ^ns^*p* > 0.05.

### 3.6 si-SIRT3 increased mitochondrial damage and reduced mitochondrial autophagy in HT22 cells

To investigate the effect of SIRT3 on mitochondrial function in HT22 cells treated with Aβ42, Don, and Gas, we transfected the cells with SIRT3 siRNA. Subsequently, we evaluated the cell viability, ATP, ROS, and mitochondrial membrane potential levels of each group. QPCR and Western blot were used to determine the most effective transfection sequence, and the results are shown in Figures 6A–C. Sequence 1 had the highest interference efficiency (*p* < 0.0001), so this sequence was selected for the subsequent experiments. CCK8 and flow cytometry apoptosis detection results showed that the combined treatment of Don and Gas could significantly enhance the cell viability of the damaged model cells and inhibit cell apoptosis (*p* < 0.0001); after transfection with si-SIRT3, the cell protective effect of Don+Gas was significantly weakened (*p* < 0.05); at the same time, intervention with an AMPK inhibitor could also reverse the anti-apoptotic effect of Don+Gas (*p* < 0.05) Figures 6D–F. The detection of mitochondrial function indicators further confirmed (Figures 7A–D) that Don+Gas could effectively increase the cell ATP content, reduce ROS levels, and maintain stable mitochondrial membrane potential (*p* < 0.0001); while knocking down SIRT3 or inhibiting AMPK activity significantly weakened the protective effect of Don+Gas on mitochondrial function and alleviation of oxidative stress (*p* < 0.05). These experiments confirmed that blocking AMPK or silencing SIRT3 could weaken the cell protection, mitochondrial stability, and antioxidant stress effects mediated by Gas combined with Don. This reveals that Gas combined with Don may exert neuroprotective effects by activating the AMPK/PGC-1α/SIRT3 signaling axis.

**Figure 6:**
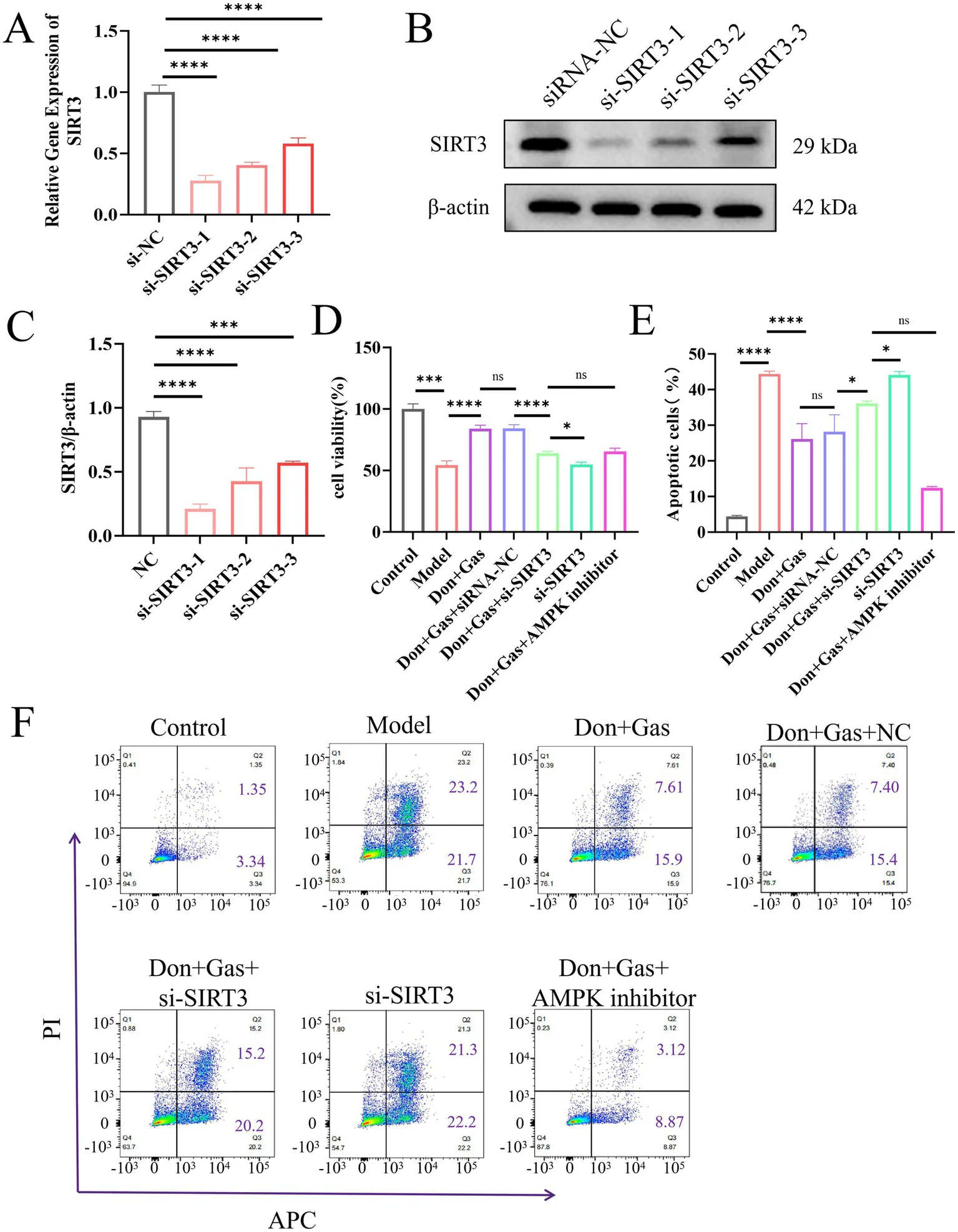
si-SIRT3 or inhibition of AMPK can reverse the protective and anti-apoptotic effects of Don combined with Gas on damaged cells. **(A)** RT-QPCR was used to detect the mRNA level of SIRT3 (*n* = 3). **(B,C)** Western blot was used to detect the protein expression level of SIRT3 (*n* = 3). **(D)** CCK8 was used to detect cell viability (*n* = 3). **(E,F)** Flow cytometry was used to detect cell apoptosis (*n* = 3). Data are presented as mean ± SEM. *p < 0.05, ***p < 0.001, ****p < 0.0001, ^ns^*p* > 0.05.

**Figure 7:**
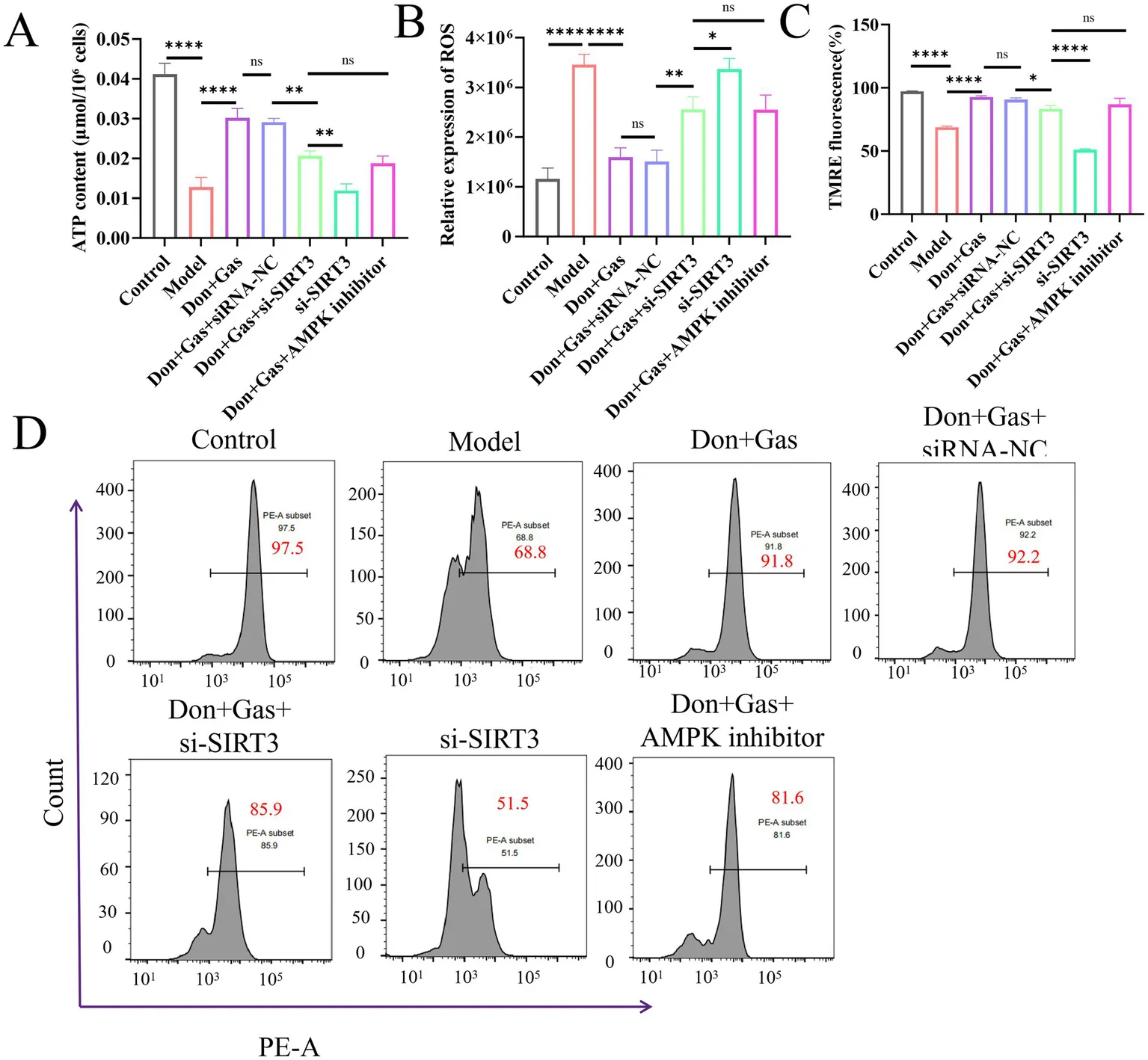
Inhibition of si-SIRT3 or AMPK can weaken the improvement effect of Don combined with Gas on mitochondrial function. **(A)** Detection of cellular ATP content (*n* = 3). **(B)** Detection of cellular ROS level using DCFH-DA fluorescent probe (*n* = 3). **(C,D)** Flow cytometry detection of mitochondrial membrane potential (*n* = 3). Data are presented as mean ± SEM. *p < 0.05, **p < 0.01, ****p < 0.0001, ^ns^*p* > 0.05.

### 3.7 si-SIRT3 or inhibition of AMPK can block the activation of the AMPK/PGC-1α/SIRT3 pathway by Gas combined with Don

To further verify the regulatory relationship of Gas combined with Don on the AMPK/PGC-1α/SIRT3 pathway, we conducted a compensation experiment and detected the expression of pathway proteins. The Western blot results (Figure 8) showed that compared with the Model group, the combined treatment of Don + Gas could significantly up-regulate the protein levels of p-AMPK/AMPK, PGC-1α and SIRT3 (*p* < 0.0001); after transfection with si-SIRT3, the expression of PGC-1α and SIRT3 significantly decreased (*p* < 0.05), but the level of p-AMPK/AMPK did not change significantly; after intervention with AMPK inhibitor, the protein expressions of p-AMPK/AMPK, PGC-1α and SIRT3 were all significantly down-regulated (*p* < 0.05).

**Figure 8:**
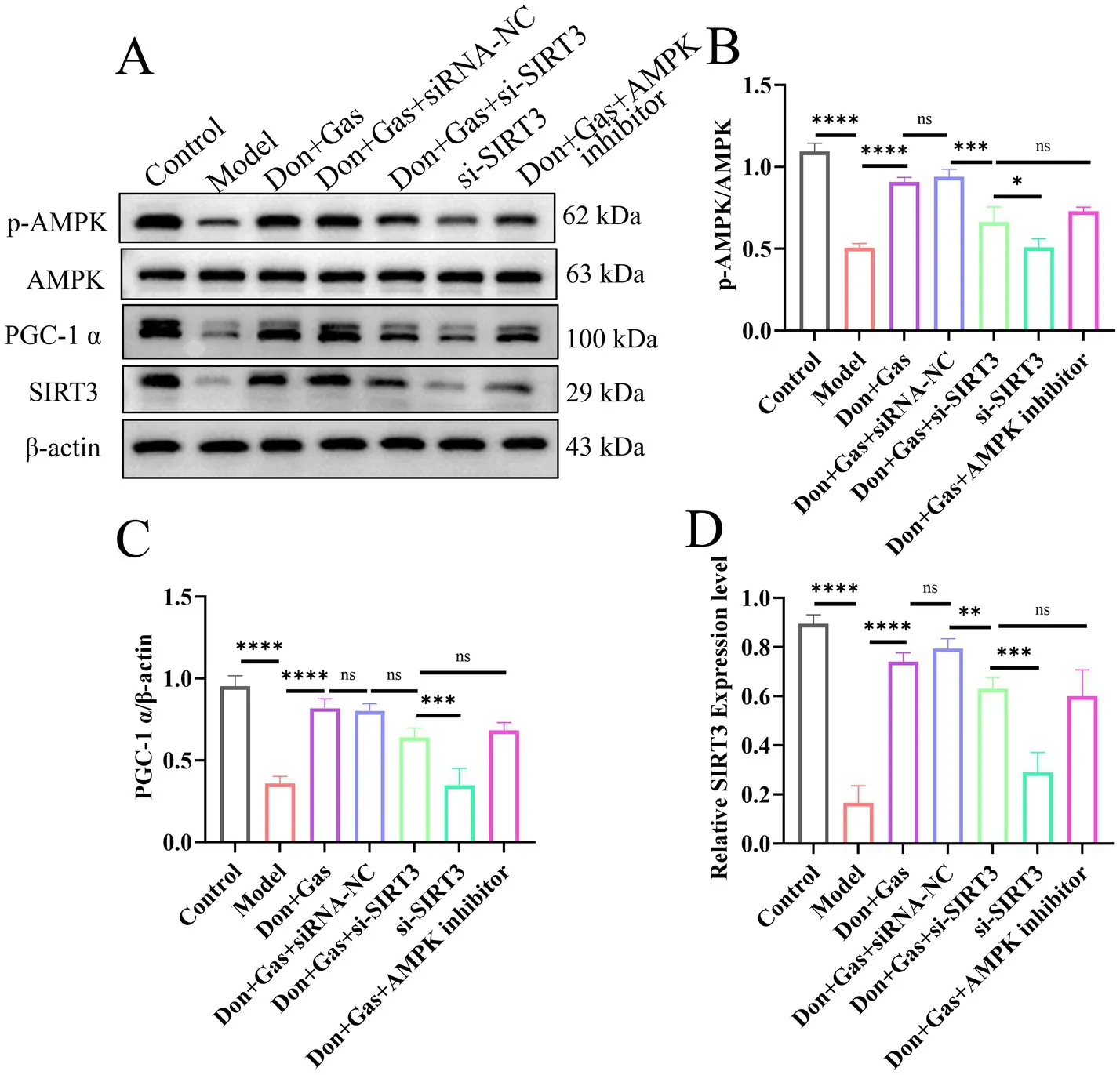
Inhibition of si-SIRT3 or AMPK can prevent the activation of the AMPK/PGC-1α/SIRT3 pathway induced by the combination of Gas and Don. **(A–D)** Western blot was used to detect the protein expression levels of p-AMPK, AMPK, PGC-1α and SIRT3 (*n* = 3). The data are presented as the mean ± standard error (SEM). *p < 0.05, **p < 0.01, ***p < 0.001, ****p < 0.0001, ^ns^*p* > 0.05.

The above results suggest that AMPK is upstream of SIRT3. Gas combined with Don activates AMPK and successively up-regulates the expression of downstream PGC-1α and SIRT3; inhibition of AMPK can block the activation of the entire pathway, and SIRT3 is a key effector molecule downstream of this pathway. This clarifies the upstream-downstream cascade relationship of AMPK/PGC-1α/SIRT3, and further confirms that Gas combined with Don may exert protective effects by activating the AMPK/PGC-1α/SIRT3 signaling axis.

## 4 Discussion

AD is a common neurodegenerative disorder characterized by Aβ deposition and excessive phosphorylation of Tau protein, resulting in irreversible neurological damage (Detka et al., 2024; Courade et al., 2025; Long et al., 2024; Luo et al., 2025; Yang et al., 2024). The first-line clinical drugs such as Don can only temporarily alleviate cognitive symptoms but cannot halt the progression of the disease and have certain side effects and medication risks (Zhu et al., 2024). Therefore, a multi-target combined treatment strategy of Chinese and Western medicines that combines efficacy and safety has become a new research direction for the treatment of AD. Gas, the main active component of *Gastrodia elata*, has few side effects and can exert anti-AD effects through antioxidant and mitochondrial function improvement pathways (Wang et al., 2024; Wang et al., 2024; Li B. et al., 2025; Wang J. et al., 2026; Fasina et al., 2022). At the same time, Gas can improve mitochondrial function by regulating SIRT3 (Chen et al., 2024; Zhang et al., 2023). However, the efficacy of Gas as a single drug is mild and its targeted regulatory ability is limited. Don, in addition to the classic cholinergic regulatory effect (Wang C. et al., 2026; Wu et al., 2021; Li Y. et al., 2025), can also activate the AMPK pathway to promote mitochondrial biogenesis (Abu-Risha et al., 2024; Cho et al., 2025). Don has important protective effects on ischemia–reperfusion injury, secondary neurological injury after subarachnoid hemorrhage, and mitochondrial dysfunction caused by circadian rhythm disorders (Khuanjing et al., 2021; Rehman et al., 2025; Kenche et al., 2021). These studies suggest that Gas, which maintains mitochondrial homeostasis through the SIRT3 pathway, may have complementary mechanisms. However, currently, both are mostly studied separately, and the efficacy and molecular mechanism of combined intervention in AD still need to be clarified.

In APP/PS1 double transgenic mice, Aβ deposition in the brain began to gradually appear at the age of 4 to 6 months, reaching a peak at 9 to 12 months (Tag et al., 2022). We used 6-month-old mice and collected samples at 8 months of age to investigate the neuroprotective effects of Gas in combination with Don. *In vivo* behavioral results confirmed that Gas combined with Don could further improve spatial memory, working memory and object recognition memory in APP/PS1 mice. These results suggest that Gas combined with Don may play a neuroprotective effect in the progressive stage of Aβ deposition and effectively delay cognitive decline.

Mitochondrial dysfunction has been proven to be a key driving event in the early stage of AD onset (D'Alessandro et al., 2025). Mitochondria are the core organelles of ROS generation and oxidative stress regulation in neurons. Mitochondrial dysfunction and abnormal accumulation of ROS form a vicious cycle, which is an important mechanism mediating the pathogenesis of AD (Klemmensen et al., 2024). Due to the vigorous metabolism and weak antioxidant capacity of neurons, it is easy to cause excessive accumulation of ROS due to leakage of mitochondrial electron transport chain and induce oxidative stress damage (Ionescu-Tucker and Cotman, 2021). Excessive ROS will continue to damage the mitochondrial structure and respiratory chain function, inhibit oxidative phosphorylation and ATP production, and further aggravate mitochondrial damage, forming a vicious cycle and continuously promoting the pathological progress of AD (Ionescu-Tucker and Cotman, 2021; Massaro et al., 2025). The AMPK/PGC-1α/SIRT3 signaling axis is a core pathway that regulates mitochondrial biosynthesis, oxidative metabolism, and antioxidant defense (Wang et al., 2025; Wang et al., 2022; Han et al., 2025). AMPK, as an energy sensor, activates and upregulates PGC-1α, further promoting the transcription of mitochondrial-related genes; the mitochondrial localization protein SIRT3 can stabilize the respiratory chain complex through deacetylation modification, eliminate ROS, and maintain mitochondrial integrity, exerting important neuroprotective effects in the central nervous system (Zhang H. et al., 2025).

The results of pathological staining of mouse brain tissue in this study further confirmed that the combined treatment could further reduce Aβ plaque deposition in the cortex and hippocampus and activate AMPK/PGC-1α/SIRT3 signaling axis to inhibit APP abnormal activation and Tau protein hyperphosphorylation. This finding was corroborated by several recent studies (D'Alessandro et al., 2025; Han et al., 2025). Studies have shown that Gas mainly improves mitochondrial function by upregulating SIRT3 expression and activity (Chen et al., 2024; Zhou et al., 2026), while Don mainly initiates mitochondrial biogenesis by activating AMPK phosphorylation (Kim et al., 2016). In addition, Gas can exert neuroprotective effects through various pathways such as antioxidation, anti-inflammation, regulation of autophagy, and inhibition of ferroptosis (Wang et al., 2024; Li B. et al., 2025; Wang J. et al., 2026; Fasina et al., 2022; Gong et al., 2025). Don also improves synaptic transmission in the mouse brain through the cholinergic system (Cuello, 2025). The *in vivo* study mainly found that Gas combined with Don could further improve mitochondrial dysfunction. Based on the above research background and experimental results, it is suggested that Gas combined with Don may have an important effect on a more comprehensive intervention of multiple pathological aspects of AD. However, the alleviation of anti-inflammation and antioxidant *in vivo* deserves further exploration in the future.

HT22 cells are stable in culture, amenable to pharmacological and genetic intervention, and widely used in neurotoxicity and mitochondrial function studies (Wang et al., 2025; Reddy et al., 2021). To further dissect the effects of Gas and Don on mitochondrial dysfunction, HT22 cells were used as clues to the non-cholinergic downstream mechanisms of the combined treatment on the mitochondrial axis. The results showed that Gas combined with Don further promoted cell proliferation and enhanced mitochondrial function in AD models compared with the interventions alone. When mitochondrial ATP levels increased, ROS accumulation decreased, and mitochondrial membrane potential recovered, mitochondrial function was stabilized, and neuronal cell damage was alleviated. Therefore, the mitochondrial membrane potential, ROS, and ATP levels gradually recovered, alleviating the pathological manifestations of AD.

SIRT3 is an important mitochondrial deacetylase that regulates various cellular functions through deacetylation of mitochondrial proteins, including inducing mitochondrial energy metabolism, oxidative stress, and autophagy (Xian et al., 2025; Xi et al., 2025; Dai et al., 2024). The expression of SIRT3 is decreased in AD (Dai et al., 2024), making activation of SIRT3 a potential treatment for neurodegenerative diseases. Studies have shown that enhancing the expression or activity of SIRT3 can improve mitochondrial energy metabolism in APP/PS1 transgenic mice, alleviate synaptic damage and cognitive deficits (Li et al., 2024). The PGC-1α is related to the expression of SIRT3 (Wang et al., 2025). PGC-1α indirectly controls SIRT3 transcription by promoting NRF2 and ERRα (Yao et al., 2026; Menezes et al., 2026). AMPK acts as a cellular energy sensor in response to low ATP levels by stimulating energy-saving processes (Steinberg and Hardie, 2023). At the same time, it is essential to regulate the level of oxidative stress by affecting antioxidant defense and enhancing mitochondrial biogenesis (Wang et al., 2022). It has been confirmed that Gas combined with Don could further restore AMPK phosphorylation in APP/PS1 mice brain tissue. In addition, AMPK targets various cellular proteins, such as cAMP response element binding protein (CREB), which is a well-known catalyst of PGC-1α, can increase the expression of mitochondrial SIRT3 (Zhang et al., 2020). Therefore, AMPK, PGC-1α, and SIRT3 are identified as important components of intracellular signaling pathways regulating cellular energy output and oxidative stress. This mechanism is similar to the neuroprotective mechanism of Gas combined with Don observed in AD.

In this study, the combination of Gas and Don can further reduce the production of ROS by enhancing the protective mitochondrial function. Knocking down SIRT3 and blocking the AMPK pathway leads to an increase in mitochondrial damage, and the cell viability of HT22 cells significantly decreases. At the same time, this significantly weakens the neuroprotective effect of the combination of Gas and Don. This suggests that Gas combined with Don may protect hippocampal neurons in AD mice by further activating AMPK/PGC-1α/SIRT3 axis. The multi-target effect of the combined treatment may have potential therapeutic effects in improving disease outcomes and reducing adverse events.

Although this study preliminarily revealed the neuroprotective effect of Gas combined with Don based on neuronal mitochondrial homeostasis, there are still some limitations. First, neuroinflammation mediated by activation of microglia and astrocytes was not investigated in this study, nor were they validated in human SH-SY5Y cells. Further studies are needed to perform genetic validation of AMPK *in vitro* and *in vivo*. Secondly, the experimental design and sample size need to be improved: the behavioral sample size is still slightly insufficient, and only a single dose of fixed combination is used, so it is difficult to determine whether the two drugs are additive or synergistic. In the future, the sample will be expanded, and the matching will be optimized by Chou-Talalay independence model, Bliss independence model and chessboard test to take into account the efficacy and peripheral cholinergic side effects of Don. Finally, the basis for clinical translation is still insufficient. At present, there is a lack of studies on pharmacokinetics, blood–brain barrier penetration, long-term toxicity and human dose. LC–MS/MS, allometric amplification and PBPK models are planned to evaluate the route of administration, bioavailability and brain delivery efficiency, so as to provide a basis for clinical dose optimization.

## 5 Conclusion

In summary, this study has demonstrated that Gas combined with Don can activate the AMPK/PGC-1α/SIRT3 signaling axis, improve the mitochondrial function of neurons, inhibit Aβ plaque deposition and excessive phosphorylation of Tau protein, and may exert certain beneficial effects on cognitive impairment in APP/PS1 mice. This study can provide preliminary experimental clues for the joint intervention of Gas and Don in Alzheimer’s disease, and may offer useful background information for preclinical research into combined treatment strategies for AD.

## Data Availability

The original contributions presented in the study are included in the article/Supplementary material, further inquiries can be directed to the corresponding authors.

## References

[ref1] Abu-Risha S. E. Sokar S. S. Elzorkany K. E. Elsisi A. E. (2024). Donepezil and quercetin alleviate valproate-induced testicular oxidative stress, inflammation and apoptosis: imperative roles of AMPK/SIRT1/ PGC-1α and p38-MAPK/NF-κB/ IL-1β signaling cascades. Int. Immunopharmacol. 134:112240. doi: 10.1016/j.intimp.2024.112240, 38744177

[ref2] Angelova V. T. Stoyanov B. P. Simeonova R. (2024). New insights into the development of donepezil-based hybrid and natural molecules as multi-target drug agents for Alzheimer's disease treatment. Molecules 29:5314. doi: 10.3390/molecules29225314, 39598703 PMC11596391

[ref3] Chen Y. X. Yang H. Wang D. S. Chen T. T. Qi X. L. Tao L. . (2024). Gastrodin alleviates mitochondrial dysfunction by regulating SIRT3-mediated TFAM acetylation in vascular dementia. Phytomedicine 128:155369. doi: 10.1016/j.phymed.2024.155369, 38547618

[ref4] Cho W. Choi S. W. Lim D. S. Gwon H. J. Abd el-Aty A. M. Ahmet Aydemir H. . (2025). Donepezil alleviates hepatic steatosis by mitigating ER stress via the AMPK/autophagy pathway. Mol. Cell. Endocrinol. 601:112523. doi: 10.1016/j.mce.2025.112523, 40118333

[ref5] Courade J. P. Zetterberg H. Höglinger G. U. Dewachter I. (2025). The evolving landscape of Alzheimer's disease therapy: from aβ to tau. Cell 188, 7337–7354. doi: 10.1016/j.cell.2025.11.033, 41448139

[ref6] Cuello A. C. (2025). The involvement of the cholinergic system in Alzheimer disease. Handb. Clin. Neurol. 211, 63–79. doi: 10.1016/B978-0-443-19088-9.00005-6, 40340068

[ref7] Dai N. Su X. Li A. Li J. Jiang D. Wang Y. (2024). DVL/GSK3/ISL1 pathway signaling: unraveling the mechanism of SIRT3 in neurogenesis and AD therapy. Stem Cell Res. Ther. 15:299. doi: 10.1186/s13287-024-03925-8, 39267160 PMC11395226

[ref8] D'Alessandro M. C. B. Kanaan S. Geller M. Praticò D. Daher J. P. L. (2025). Mitochondrial dysfunction in Alzheimer's disease. Ageing Res. Rev. 107:102713. doi: 10.1016/j.arr.2025.102713 40023293

[ref9] Detka J. Płachtij N. Strzelec M. Manik A. Sałat K. (2024). p38α mitogen-activated protein kinase-an emerging drug target for the treatment of Alzheimer's disease. Molecules 29:4354. doi: 10.3390/molecules29184354, 39339348 PMC11433989

[ref10] Fasina O. B. Wang J. Mo J. Osada H. Ohno H. Pan W. . (2022). Gastrodin from Gastrodia elata enhances cognitive function and neuroprotection of AD mice via the regulation of gut microbiota composition and inhibition of neuron inflammation. Front. Pharmacol. 13:814271. doi: 10.3389/fphar.2022.814271, 35721206 PMC9201506

[ref11] Gong C. Fu X. Ma Q. He M. Zhu X. Liu L. . (2025). Gastrodin: modulating the xCT/GPX4 and ACSL4/LPCAT3 pathways to inhibit ferroptosis after ischemic stroke. Phytomedicine 136:156331. doi: 10.1016/j.phymed.2024.156331, 39731833

[ref12] Gu C. Kong F. Zeng J. Geng X. Sun Y. Chen X. (2023). Remote ischemic preconditioning protects against spinal cord ischemia-reperfusion injury in mice by activating NMDAR/AMPK/PGC-1α/SIRT3 signaling. Cell Biosci. 13:57. doi: 10.1186/s13578-023-00999-4, 36927808 PMC10018930

[ref13] Han X. Peng M. Wang X. Hu Y. Li G. Song Y. . (2025). Role of the AMPK/PGC-1α/SIRT3-mediated mitochondrial dysfunction in the neurotoxicity of methanol. Mol. Neurobiol. 63:134. doi: 10.1007/s12035-025-05431-1, 41273494

[ref14] Ionescu-Tucker A. Cotman C. W. (2021). Emerging roles of oxidative stress in brain aging and Alzheimer's disease. Neurobiol. Aging 107, 86–95. doi: 10.1016/j.neurobiolaging.2021.07.014, 34416493

[ref9001] Jiang S Li H Zhang L Mu W Zhang Y Chen T . (2025). Generic Diagramming Platform (GDP): a comprehensive database of high-quality biomedical graphics. Nucleic Acids Res. 53, D1670–D1676. doi:10.1093/nar/gkae973, 39470721 PMC11701665

[ref15] Kenche H. Singh M. Smith J. Shen K. (2021). Neuronal mitochondrial dysfunction in a cellular model of circadian rhythm disruption is rescued by donepezil. Biochem. Biophys. Res. Commun. 567, 56–62. doi: 10.1016/j.bbrc.2021.06.029, 34144501

[ref16] Khuanjing T. Palee S. Kerdphoo S. Jaiwongkam T. Anomasiri A. Chattipakorn S. C. . (2021). Donepezil attenuated cardiac ischemia/reperfusion injury through balancing mitochondrial dynamics, mitophagy, and autophagy. Transl. Res. 230, 82–97. doi: 10.1016/j.trsl.2020.10.010, 33137536

[ref17] Kim J. Lee H. J. Park S. K. Park J. H. Jeong H. R. Lee S. . (2021). Donepezil regulates LPS and aβ-stimulated neuroinflammation through MAPK/NLRP3 Inflammasome/STAT3 signaling. Int. J. Mol. Sci. 22:10637. doi: 10.3390/ijms221910637, 34638977 PMC8508964

[ref18] Kim E. Park M. Jeong J. Kim H. Lee S. K. Lee E. . (2016). Cholinesterase inhibitor donepezil increases mitochondrial biogenesis through AMP-activated protein kinase in the Hippocampus. Neuropsychobiology 73, 81–91. doi: 10.1159/000441522, 27002982

[ref19] Kimura A. M. Tsuji M. Yasumoto T. Mori Y. Oguchi T. Tsuji Y. . (2021). Myricetin prevents high molecular weight Aβ1-42 oligomer-induced neurotoxicity through antioxidant effects in cell membranes and mitochondria. Free Radic. Biol. Med. 171, 232–244. doi: 10.1016/j.freeradbiomed.2021.05.019 34015458

[ref20] Klemmensen M. M. Borrowman S. H. Pearce C. Pyles B. Chandra B. (2024). Mitochondrial dysfunction in neurodegenerative disorders. Neurotherapeutics 21:e00292. doi: 10.1016/j.neurot.2023.10.002, 38241161 PMC10903104

[ref21] Lei H. P. Yang X. Hu Y. T. Wu L. N. Wei A. H. Yu L. . (2026). Gastrodin alleviates mitochondrial energy metabolism dysfunction via activating β-catenin/c-Myc/MCT2 signaling in Alzheimer's disease models. J. Ethnopharmacol. 354:120548. doi: 10.1016/j.jep.2025.120548 40915373

[ref22] Li Y. Li L. Wang Y. Li X. Ding X. Li L. . (2025). Cholinergic signaling to CA1 astrocytes controls fear extinction. Sci. Adv. 11:eads7191. doi: 10.1126/sciadv.ads7191 PMC11970469 40184457

[ref23] Li H. Sun J. Wu Y. Yang Y. Zhang W. Tian Y. (2024). Honokiol relieves hippocampal neuronal damage in Alzheimer's disease by activating the SIRT3-mediated mitochondrial autophagy. CNS Neurosci. Ther. 30:e14878. doi: 10.1111/cns.14878, 39097923 PMC11298204

[ref24] Li B. Wang L. Xiao Y. Wang Y. Wang Y. Peng Y. . (2025). Gastrodin ameliorates tau pathology and BBB dysfunction in 3xTg-AD transgenic mice by regulating the ADRA1/NF-κB/NLRP3 pathway to reduce Neuroinflammation. Phytother. Res. 39, 1996–2016. doi: 10.1002/ptr.8461, 39963073

[ref25] Long Q. Li T. Zhu Q. He L. Zhao B. (2024). SuanZaoRen decoction alleviates neuronal loss, synaptic damage and ferroptosis of AD via activating DJ-1/Nrf2 signaling pathway. J. Ethnopharmacol. 323:117679. doi: 10.1016/j.jep.2023.117679 38160863

[ref26] Luo B. Cheng T. Xiang Y. Sun S. Liu Y. Yan J. . (2025). Promoting retinal ganglion cell regeneration with targeted liposome-based delivery of MHY1485 for optic nerve repair. J. Control. Release 383:113778. doi: 10.1016/j.jconrel.2025.113778 40288495

[ref27] Massaro M. Baudo G. Lee H. Liu H. Blanco E. (2025). Nuclear respiratory factor-1 (NRF1) induction drives mitochondrial biogenesis and attenuates amyloid beta-induced mitochondrial dysfunction and neurotoxicity. Neurotherapeutics 22:e00513. doi: 10.1016/j.neurot.2024.e00513, 39730291 PMC12014405

[ref28] Menezes E. S. Islam H. Arhen B. B. Wu Z. Pacitti L. J. Lyrae Silva N. M. . (2026). Regulation of PERM1 and select target genes in human skeletal muscle following fasting and exercise. Appl. Physiol. Nutr. Metab. 51, 1–15. doi: 10.1139/apnm-2025-0401, 41759080

[ref29] Nowell J. Blunt E. Gupta D. Edison P. (2023). Antidiabetic agents as a novel treatment for Alzheimer's and Parkinson's disease. Ageing Res. Rev. 89:101979. doi: 10.1016/j.arr.2023.101979, 37328112

[ref30] Reddy A. P. Yin X. Sawant N. Reddy P. H. (2021). Protective effects of antidepressant citalopram against abnormal APP processing and amyloid beta-induced mitochondrial dynamics, biogenesis, mitophagy and synaptic toxicities in Alzheimer's disease. Hum. Mol. Genet. 30, 847–864. doi: 10.1093/hmg/ddab054, 33615359 PMC8355469

[ref31] Rehman A. S. Tasleem A. Ali N. Khan R. Salman M. Kumar P. . (2025). Donepezil improves PSD95 expression, mitigates neuroinflammation via PI3K/Akt/NF-κB and mitochondrial dysfunction in a rodent model of subarachnoid haemorrhage. J. Neurochem. 169:e70063. doi: 10.1111/jnc.70063, 40230034

[ref32] Ren J. Xiang B. Song L. René D. J. Luo Y. Wen G. . (2024). Kaixinsan regulates neuronal mitochondrial homeostasis to improve the cognitive function of Alzheimer's disease by activating CaMKKβ-AMPK-PGC-1α signaling axis. Phytomedicine 135:156170. doi: 10.1016/j.phymed.2024.156170, 39520951

[ref33] Sanjay Sood R. Jaiswal V. Kang S. U. Park M. Lee H. J. (2024). Nobiletin regulates intracellular Ca2+ levels via IP3R and ameliorates neuroinflammation in aβ 1-42-induced astrocytes. Redox Biol. 73:103197. doi: 10.1016/j.redox.2024.103197 PMC11145555 38781730

[ref34] Steinberg G. R. Hardie D. G. (2023). New insights into activation and function of the AMPK. Nat. Rev. Mol. Cell Biol. 24, 255–272. doi: 10.1038/s41580-022-00547-x, 36316383

[ref35] Sun X. Y. Li L. J. Dong Q. X. Zhu J. Huang Y. R. Hou S. J. . (2021). Rutin prevents tau pathology and neuroinflammation in a mouse model of Alzheimer's disease. J. Neuroinflammation 18:131. doi: 10.1186/s12974-021-02182-3, 34116706 PMC8196535

[ref36] Tag S. H. Kim B. Bae J. Chang K. A. Im H. I. (2022). Neuropathological and behavioral features of an APP/PS1/MAPT (6xTg) transgenic model of Alzheimer's disease. Mol. Brain 15:51. doi: 10.1186/s13041-022-00933-8, 35676711 PMC9175339

[ref37] Wang Y. Bai M. Wang X. Peng Z. Cai C. Xi J. . (2024). Gastrodin: a comprehensive pharmacological review. Naunyn Schmiedeberg's Arch. Pharmacol. 397, 3781–3802. doi: 10.1007/s00210-023-02920-9, 38165423

[ref38] Wang D. Cao L. Zhou X. Wang G. Ma Y. Hao X. . (2022). Mitigation of honokiol on fluoride-induced mitochondrial oxidative stress, mitochondrial dysfunction, and cognitive deficits through activating AMPK/PGC-1α/Sirt3. J. Hazard. Mater. 437:129381. doi: 10.1016/j.jhazmat.2022.129381 35752048

[ref39] Wang J. Chen T. Lin Y. Shen X. Zhou M. (2026). Gastrodin and gastrodigenin as potential neuroprotectors for the treatment of neurodegenerative diseases. Am. J. Chin. Med. 54, 529–554. doi: 10.1142/s0192415x26500199, 41717900

[ref40] Wang C. Chen L. Wen R. Peng L. Chen C. Zhuo R. . (2026). Pterostilbene mitigates scopolamine-induced cognitive impairment by modulating cholinergic pathway and reducing neuroinflammation. Phytomedicine 150:157630. doi: 10.1016/j.phymed.2025.157630, 41353883

[ref41] Wang Y. Ge Y. Hua S. Shen C. Cai B. Zhao H. (2025). Aloe-Emodin improves Mitophagy in Alzheimer's disease via activating the AMPK/PGC-1α/SIRT3 signaling pathway. CNS Neurosci. Ther. 31:e70346. doi: 10.1111/cns.70346, 40125832 PMC11931456

[ref42] Wang Z. J. Li X. R. Chai S. F. Li W. R. Li S. Hou M. . (2023). Semaglutide ameliorates cognition and glucose metabolism dysfunction in the 3xTg mouse model of Alzheimer's disease via the GLP-1R/SIRT1/GLUT4 pathway. Neuropharmacology 240:109716. doi: 10.1016/j.neuropharm.2023.109716, 37730113

[ref43] Wang W. Wang Y. Wang F. Xie G. Liu S. Li Z. . (2024). Gastrodin regulates the TLR4/TRAF6/NF-κB pathway to reduce neuroinflammation and microglial activation in an AD model. Phytomedicine 128:155518. doi: 10.1016/j.phymed.2024.155518, 38552431

[ref44] Wu D. Gao D. Yu H. Pi G. Xiong R. Lei H. . (2021). Medial septum tau accumulation induces spatial memory deficit via disrupting medial septum-hippocampus cholinergic pathway. Clin. Transl. Med. 11:e428. doi: 10.1002/ctm2.428 Erratum in: Clin Transl Med. 2025 15(2):e70234. doi: 10.1002/ctm2.70234 PMC8161512 34185417

[ref45] Xi Y. Tao K. Wen X. Feng D. Mai Z. Ding H. . (2025). SIRT3-mediated deacetylation of DRP1K711 prevents mitochondrial dysfunction in Parkinson's disease. Adv. Sci. 12:e2411235. doi: 10.1002/advs.202411235, 39976201 PMC12061286

[ref46] Xian Y. Liu B. Shen T. Yang L. Peng R. Shen H. . (2025). Enhanced SIRT3 expression restores mitochondrial quality control mechanism to reverse osteogenic impairment in type 2 diabetes mellitus. Bone Res. 13:30. doi: 10.1038/s41413-024-00399-5, 40025004 PMC11873136

[ref47] Yang Y. Wei S. Tian H. Cheng J. Zhong Y. Zhong X. . (2024). Adverse event profile of memantine and donepezil combination therapy: a real-world pharmacovigilance analysis based on FDA adverse event reporting system (FAERS) data from 2004 to 2023. Front. Pharmacol. 15:1439115. doi: 10.3389/fphar.2024.1439115, 39101151 PMC11294921

[ref48] Yao M. Wang X. Wei H. Wang L. Zhang D. Liang Z. . (2026). Components of Panax ginseng and *Rhodiola rosea* regulate mitophagy via the SIRT1/3-PGC-1α-NRF2 pathway to improve myocardial ischemia-reperfusion injury. Front. Pharmacol. 17:1716078. doi: 10.3389/fphar.2026.1716078, 41727552 PMC12920564

[ref49] Zhang H. Huang Y. Yang S. Gong F. Gu Y. Xie Q. . (2025). Improved myocardial mitochondrial energy metabolism in rats with chronic heart failure by modifying fatty acid oxidation using an extract of sand-fired aconite (Jianchang gang processing). Front. Pharmacol. 16:1600410. doi: 10.3389/fphar.2025.1600410, 41019998 PMC12461213

[ref50] Zhang Y. Jin W. Chen J. Wei S. Cai W. X. Zhong Y. J. . (2023). Gastrodin alleviates rat chondrocyte senescence and mitochondrial dysfunction through Sirt3. Int. Immunopharmacol. 118:110022. doi: 10.1016/j.intimp.2023.110022, 36933487

[ref51] Zhang Y. Liu S. Cao D. Zhao M. Lu H. Wang P. (2025). Rg1 improves Alzheimer's disease by regulating mitochondrial dynamics mediated by the AMPK/Drp1 signaling pathway. J. Ethnopharmacol. 340:119285. doi: 10.1016/j.jep.2024.119285, 39733799

[ref52] Zhang J. Xiang H. Liu J. Chen Y. He R. R. Liu B. (2020). Mitochondrial Sirtuin 3: new emerging biological function and therapeutic target. Theranostics 10, 8315–8342. doi: 10.7150/thno.45922, 32724473 PMC7381741

[ref53] Zhou F. Luo Y. Liu Y. Luo L. Jiang P. Liu Q. . (2026). Gastrodin ameliorates pain-depression comorbidity through SIRT3-dependent alleviation of oxidative stress and promotion of mitochondrial biogenesis. Mol. Neurobiol. 63:400. doi: 10.1007/s12035-026-05677-3, 41604013

[ref54] Zhu C. Wang S. Ma S. Zhang S. Wang Y. Li B. . (2024). Gastrodin reduces aβ brain levels in an Alzheimer's disease mouse model by inhibiting P-glycoprotein ubiquitination. Phytomedicine 135:156229. doi: 10.1016/j.phymed.2024.156229, 39541666

