# Supplementary material for: Gastrodin combined with donepezil alleviates cognitive dysfunction in Alzheimer’s disease mice by improving mitochondrial function through AMPK/PGC-1α/SIRT3 axis
Source: Front Aging Neurosci. 2026 Oct 27;18:1978729. doi: 10.3389/fnagi.2026.1978729 (PMC13749095; doi:10.3389/fnagi.2026.1978729)
Supplement: Supplementary file 1 [file Data_Sheet_1.PDF]

Figure 3

P-AMPK	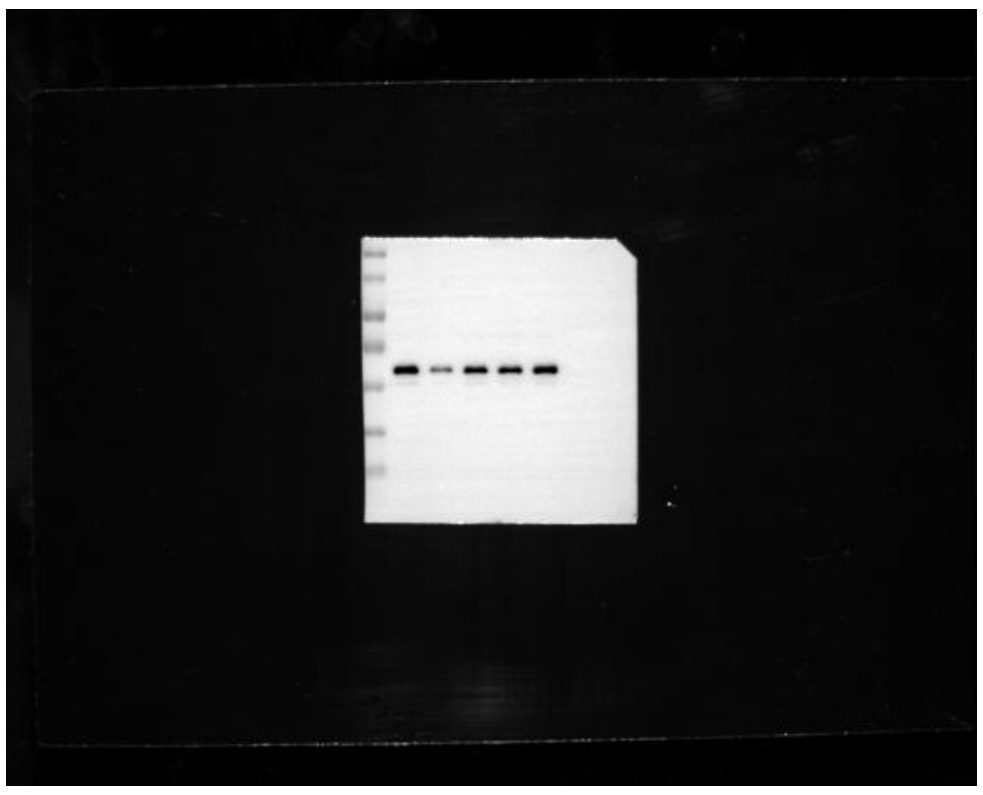
AMPK	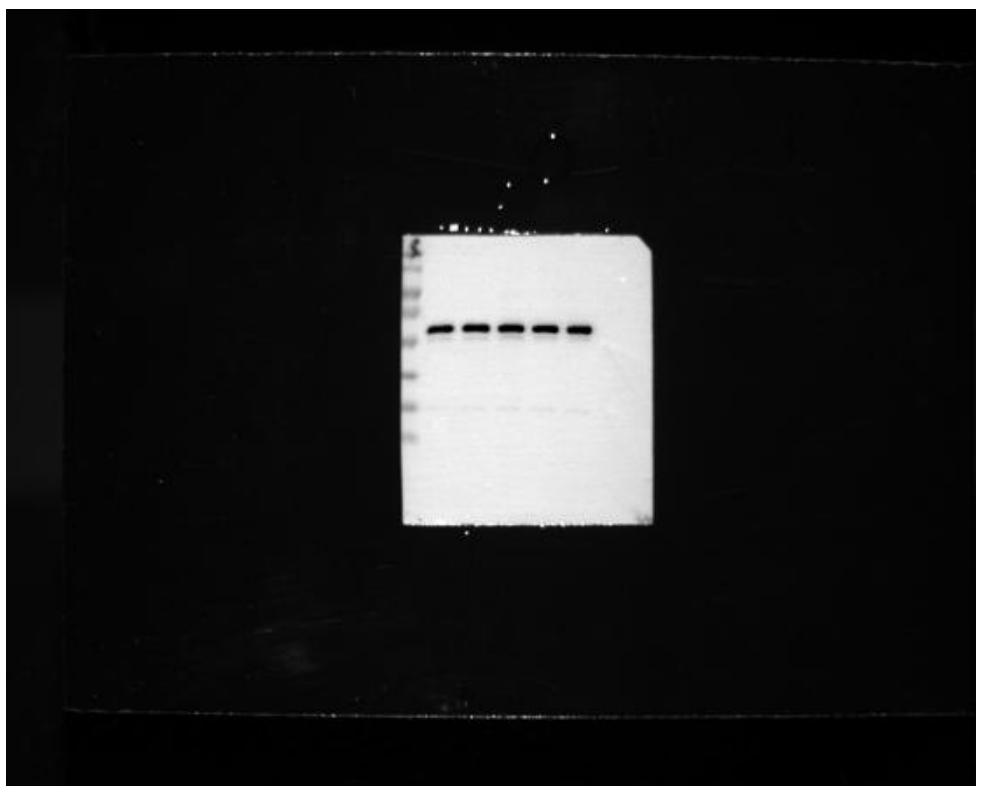
PGC-1α	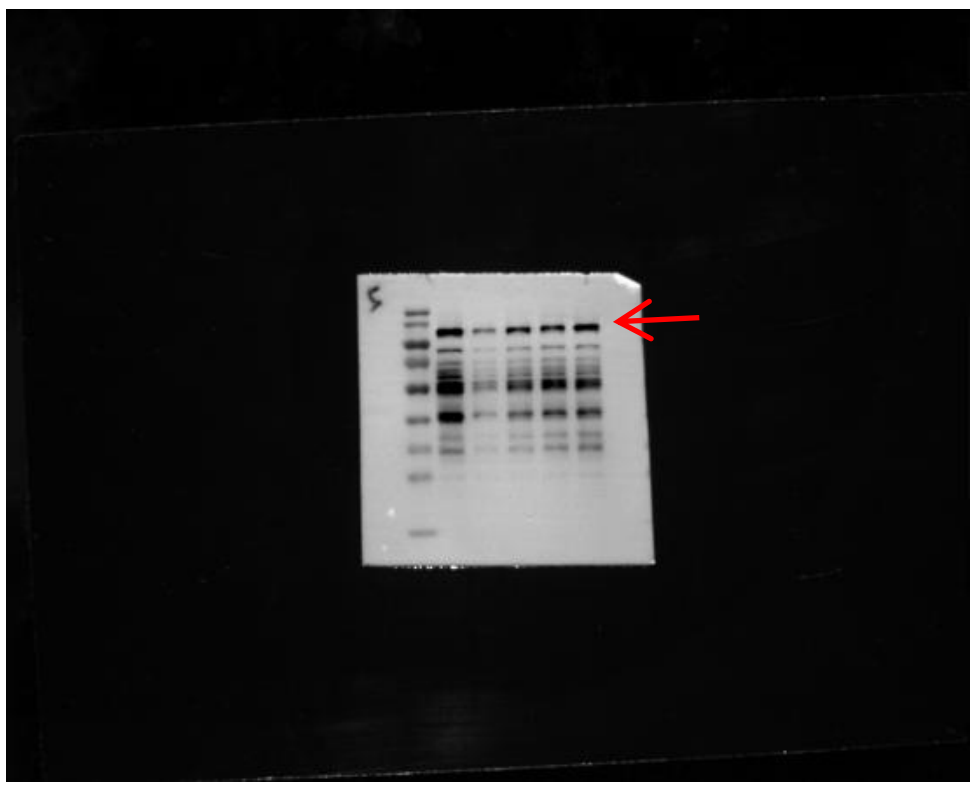
SIRT3	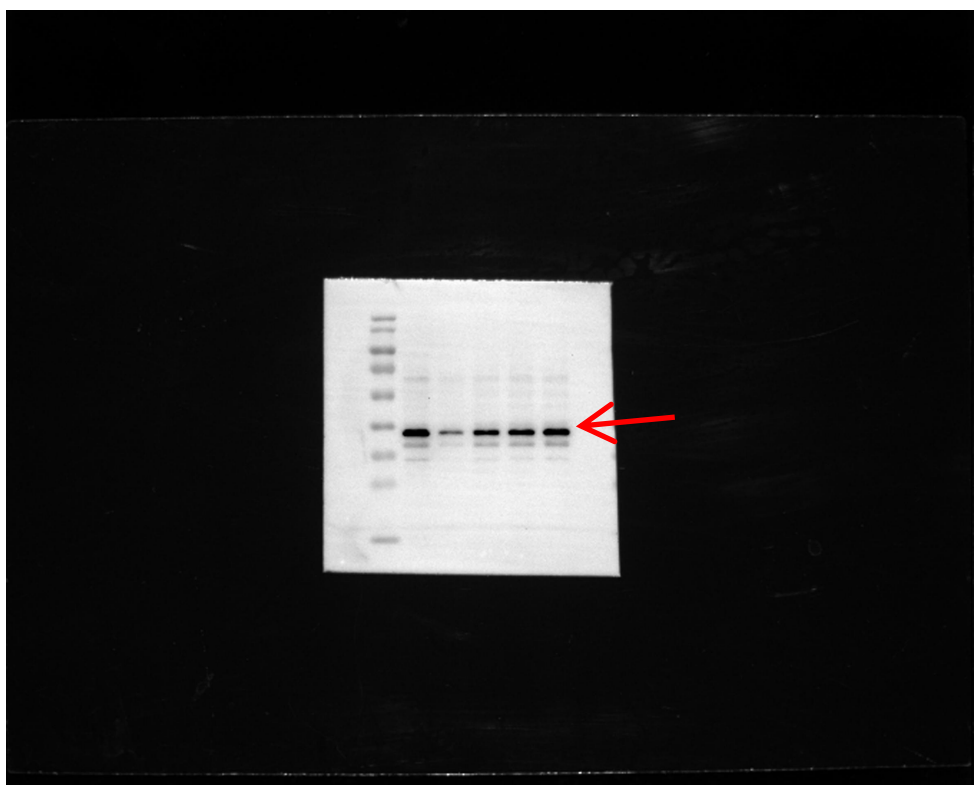

β-actin	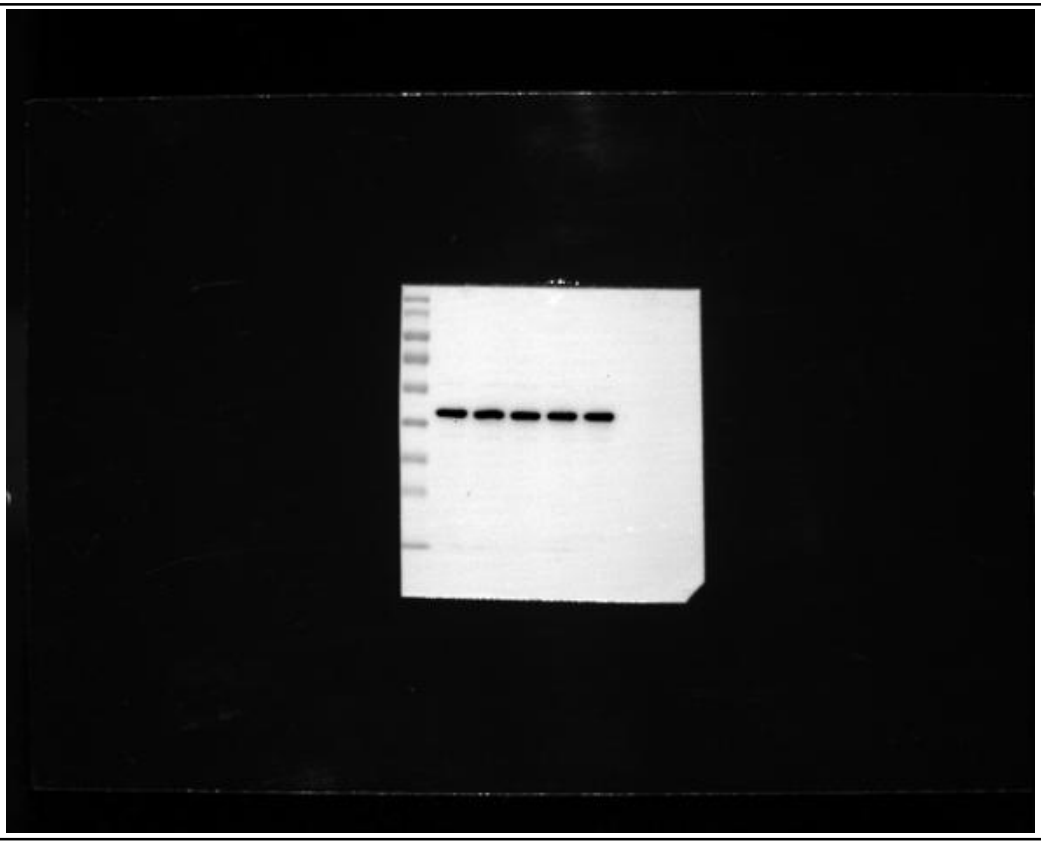
p-Tau	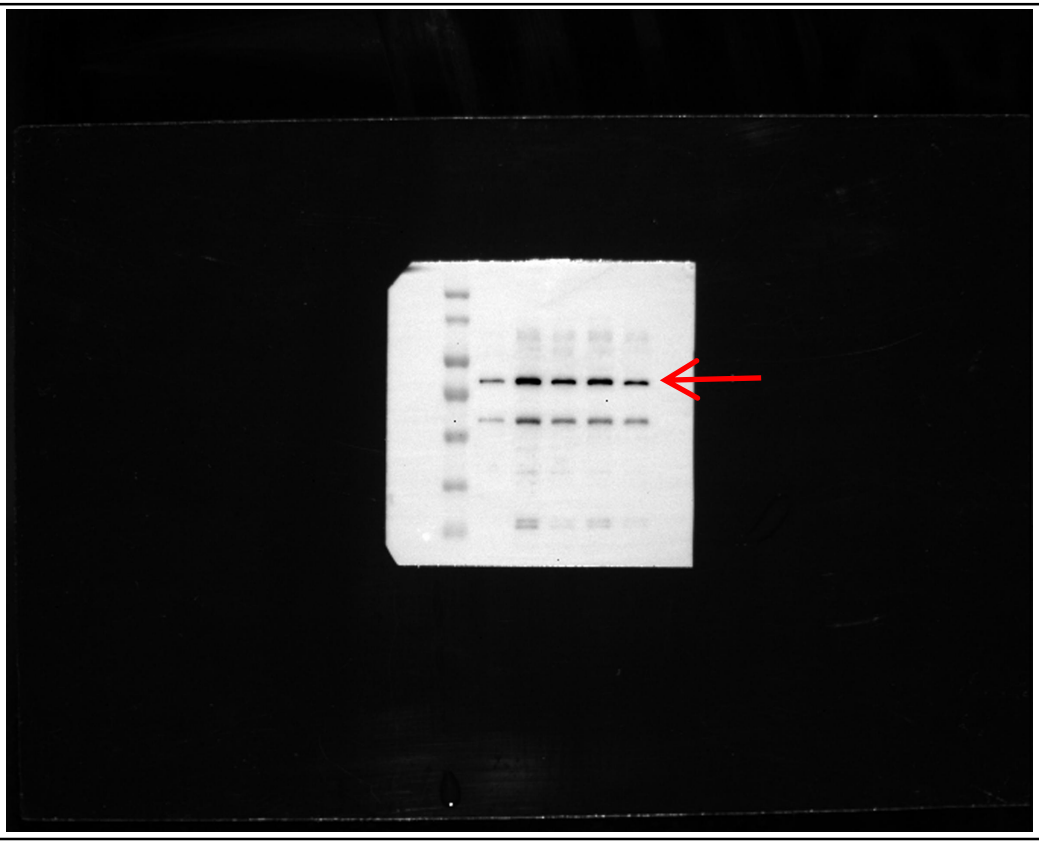
Tau	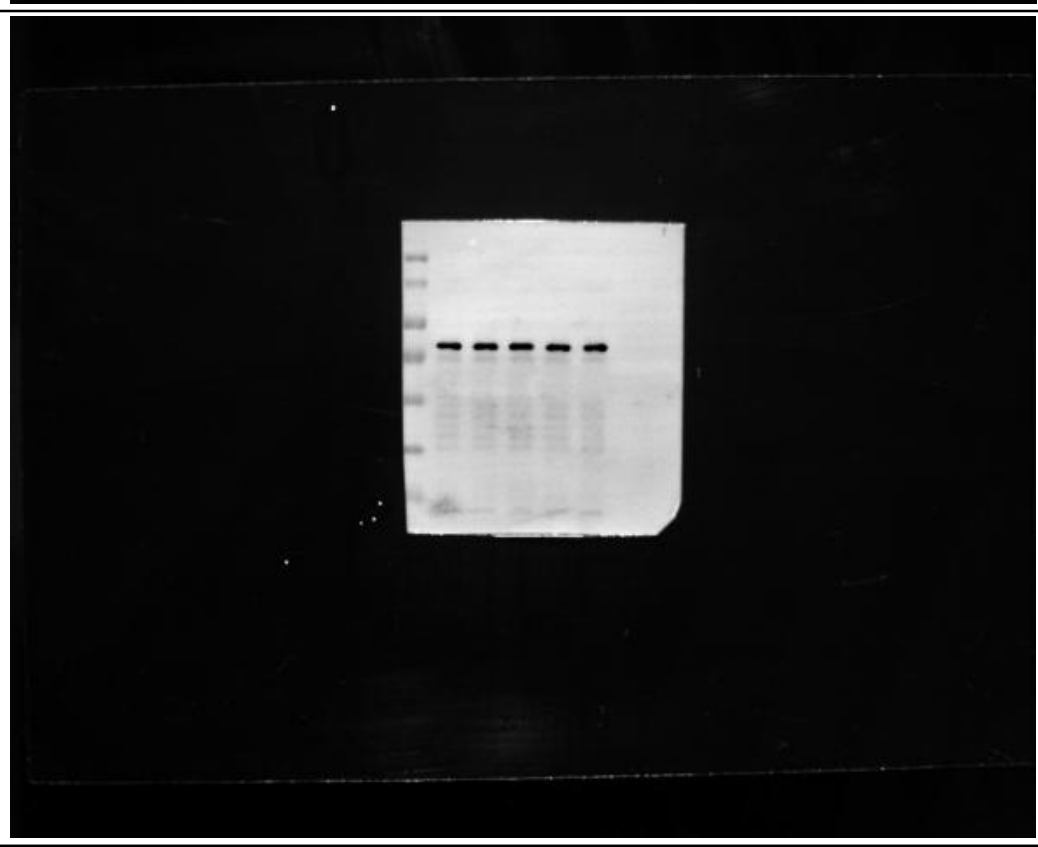
P-APP	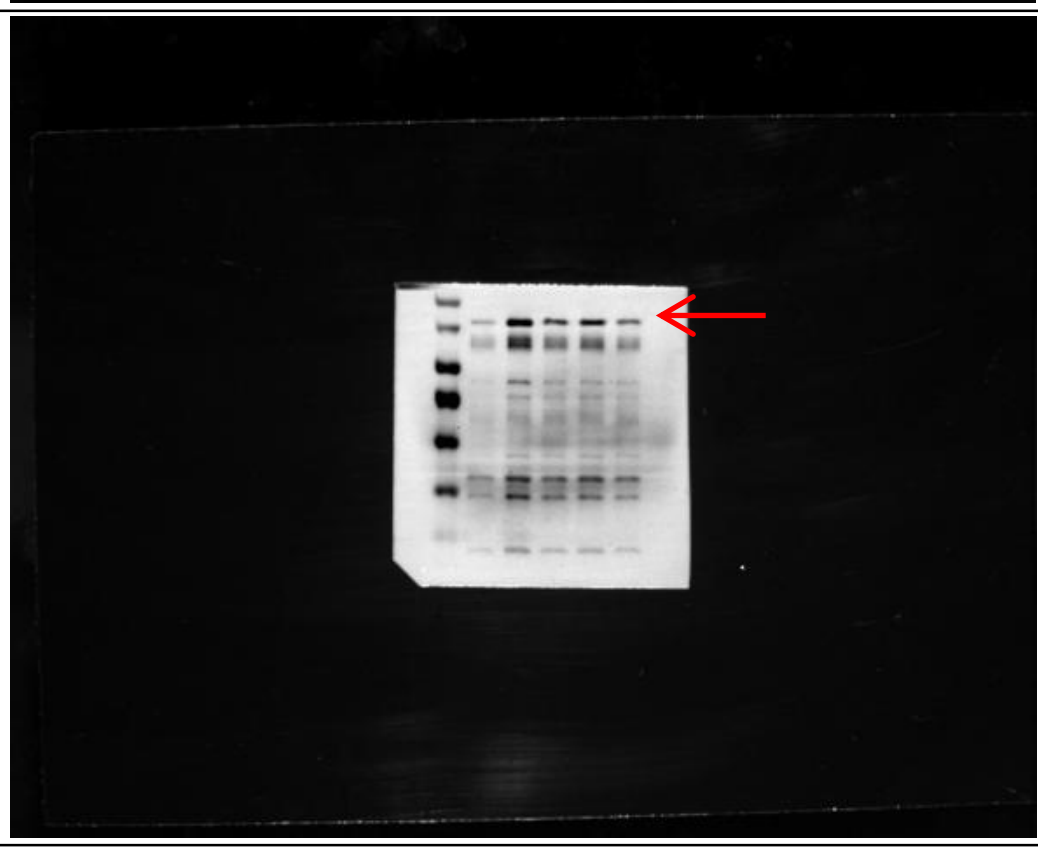

APP	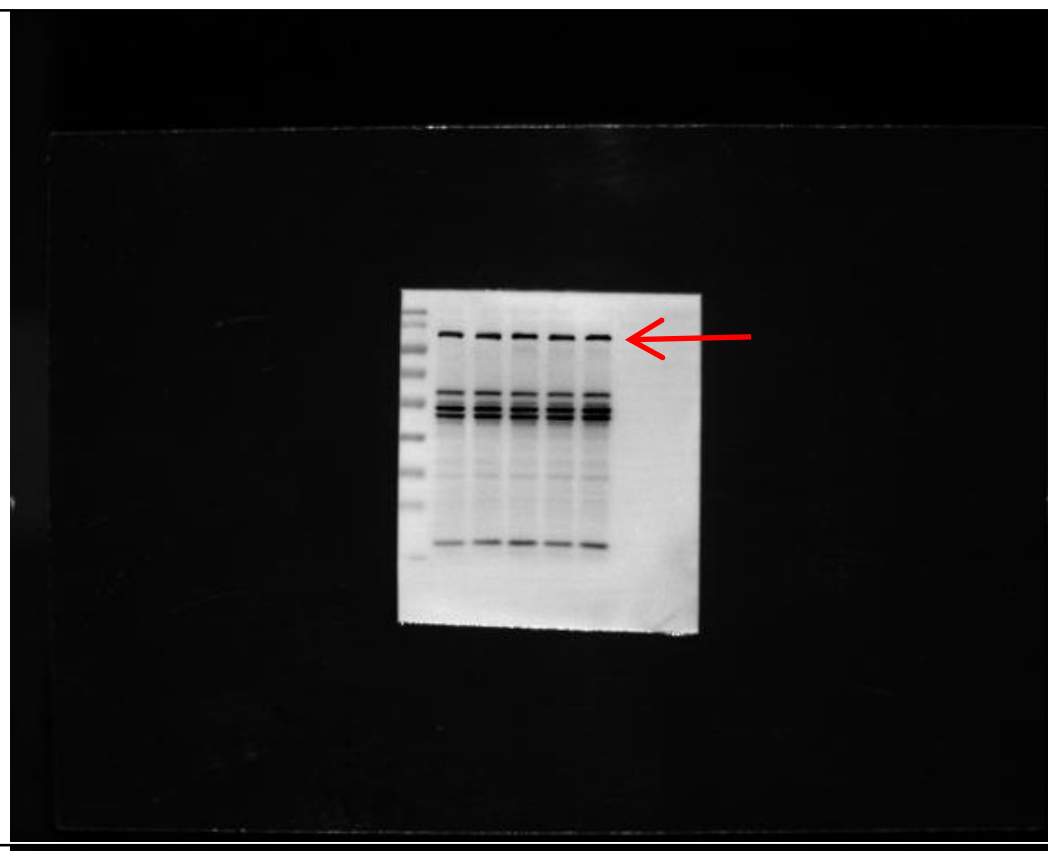
β-actin	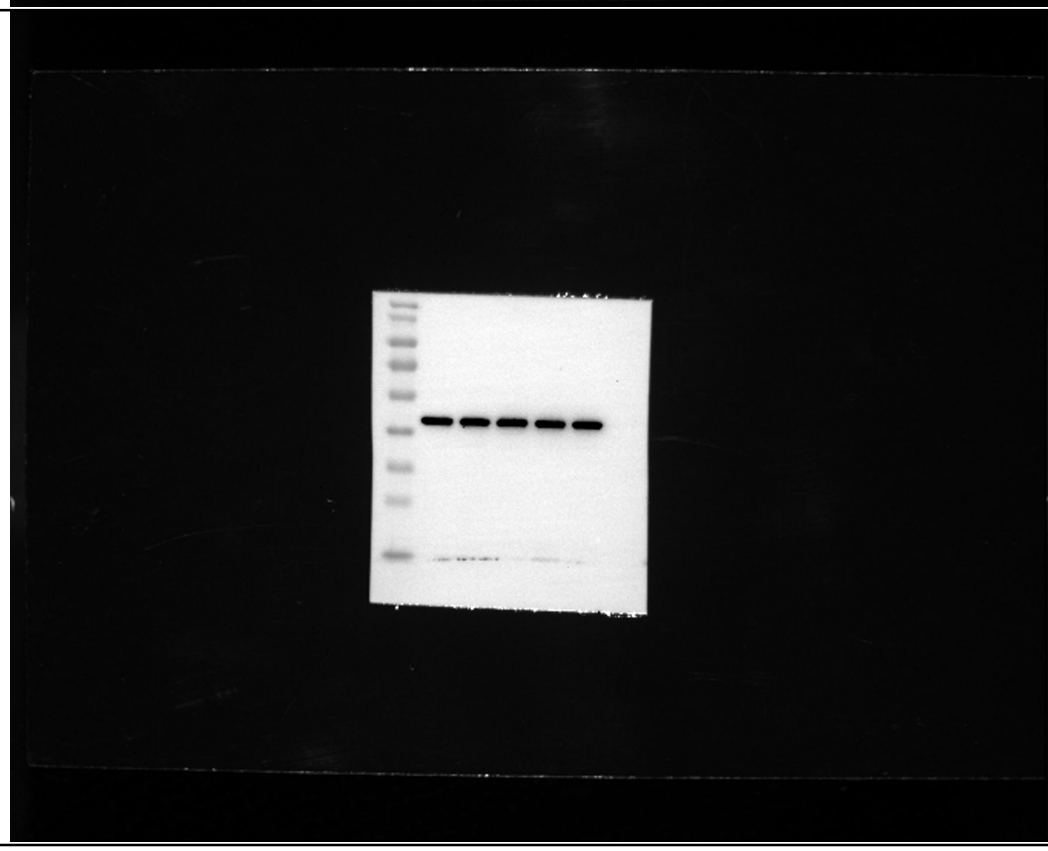

Figure 5

P-AMPK	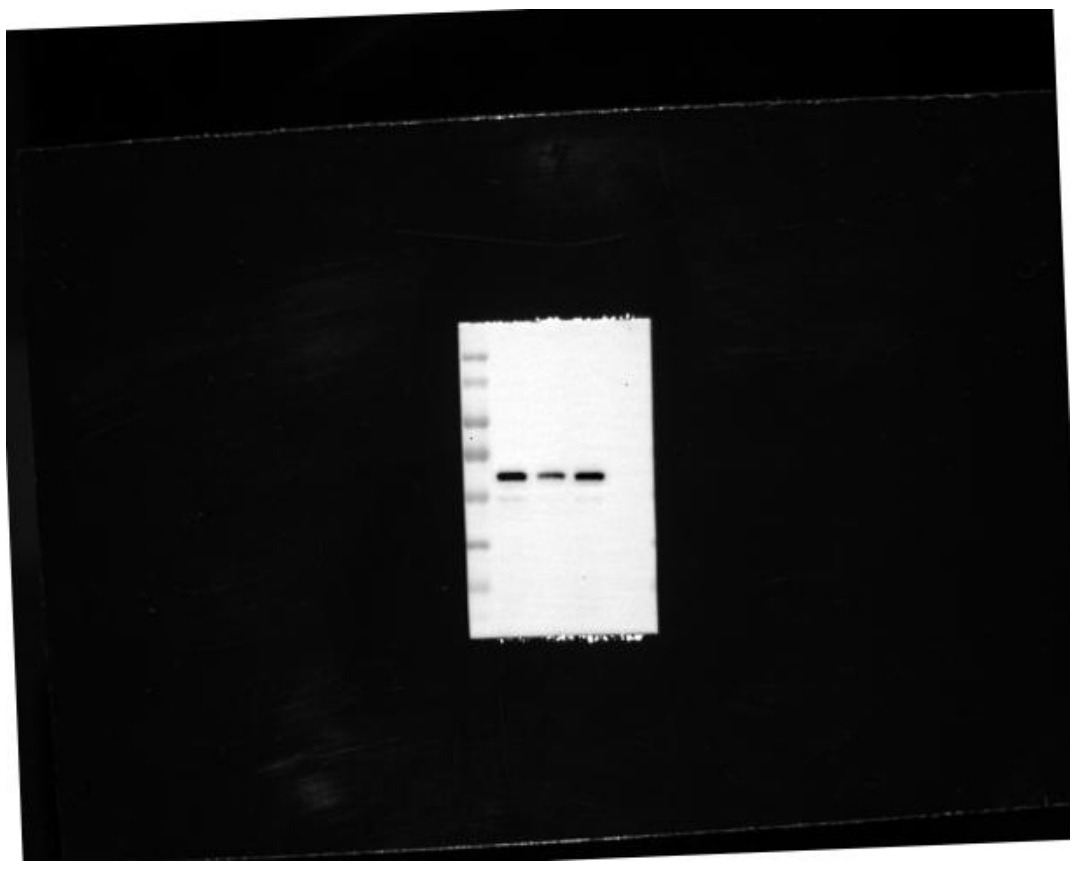

AMPK	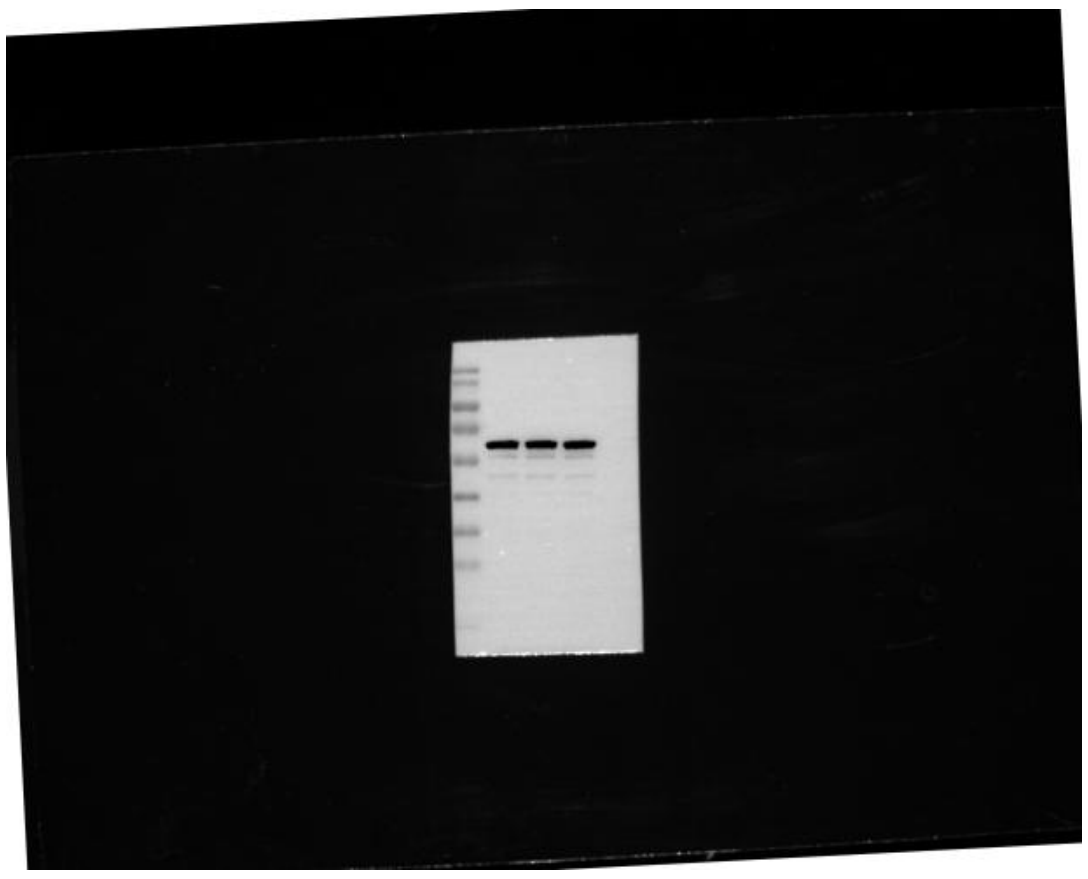
PGC-1α	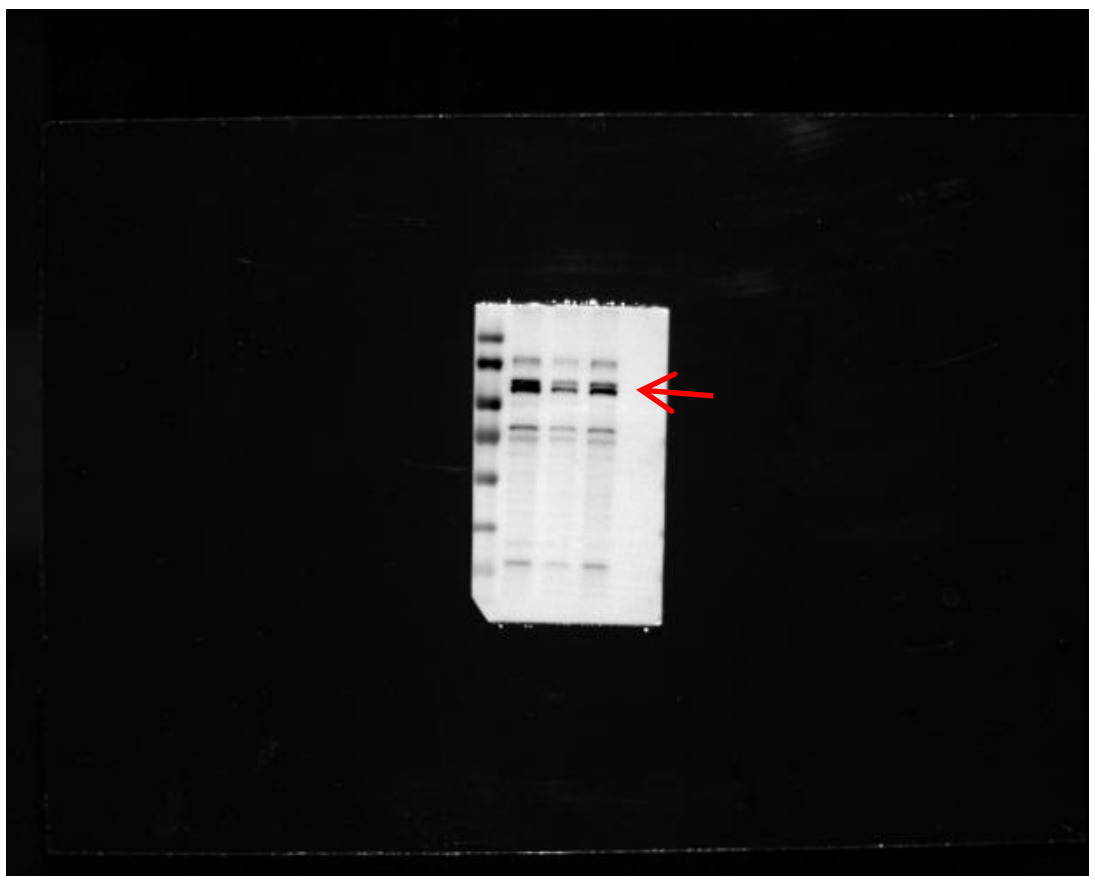
SIRT3	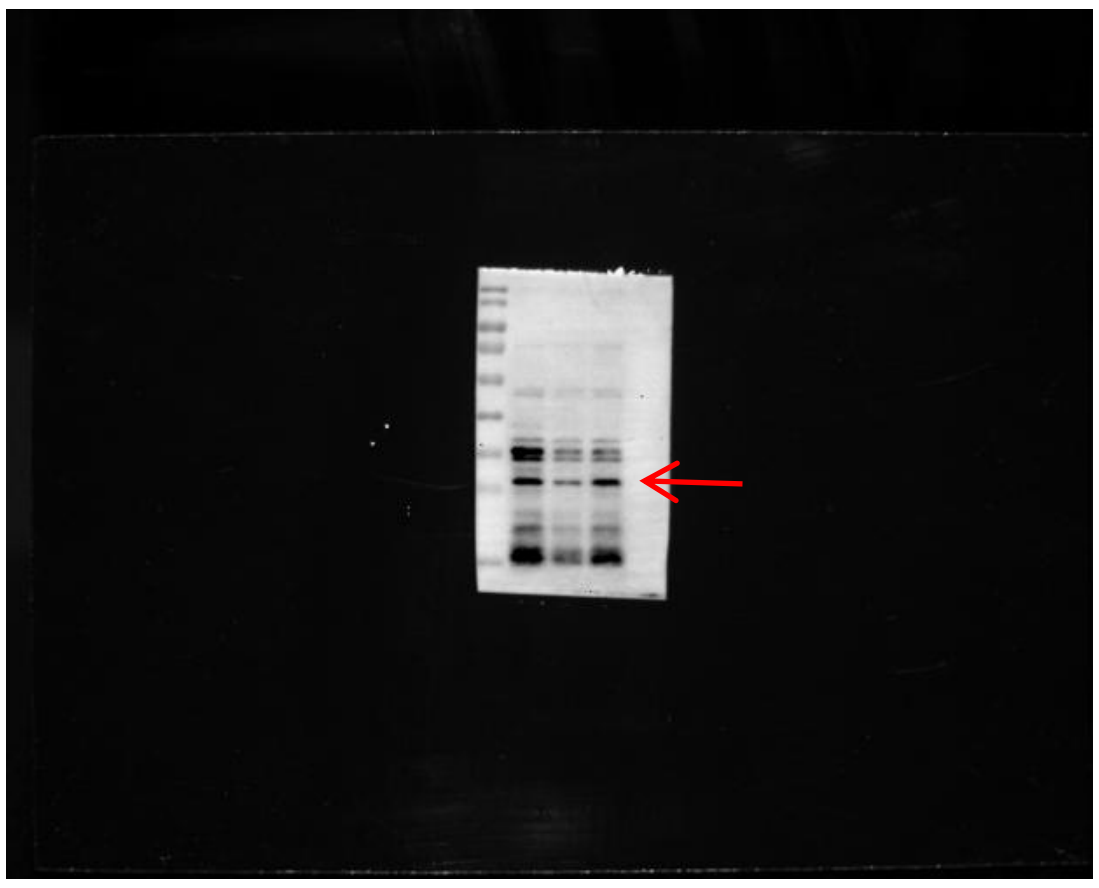
β-actin	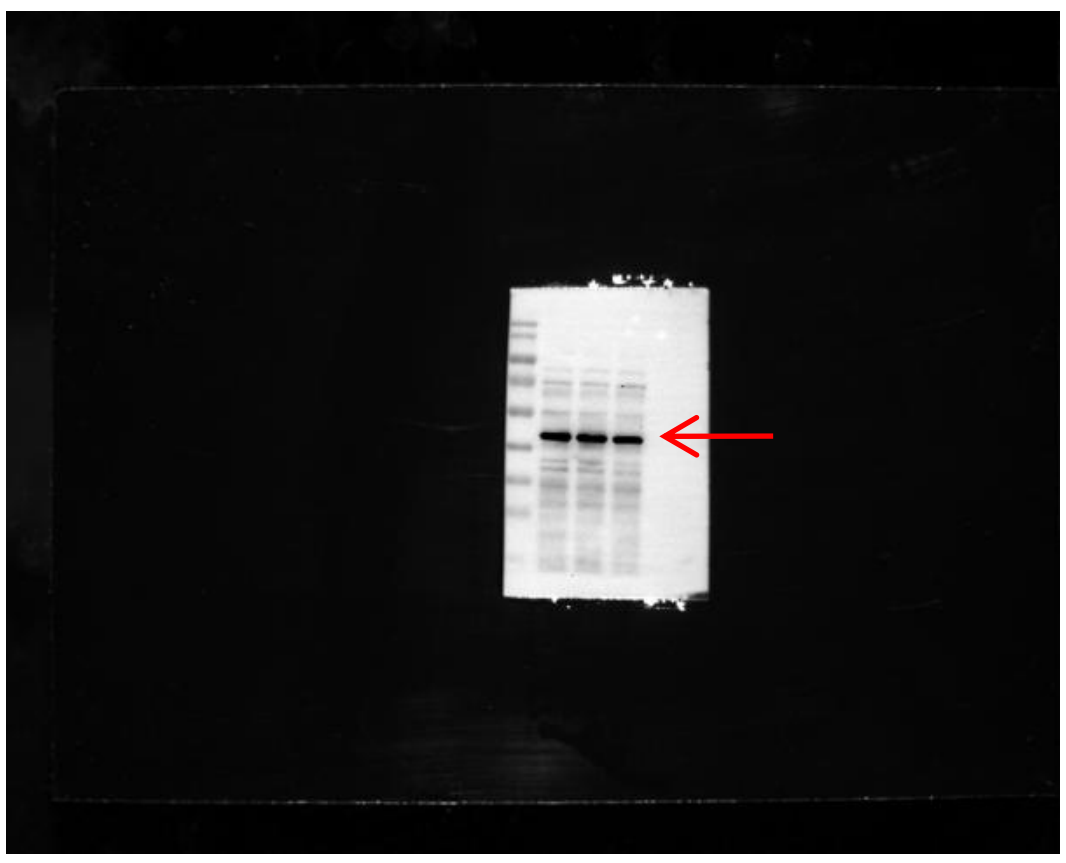

P-Tau	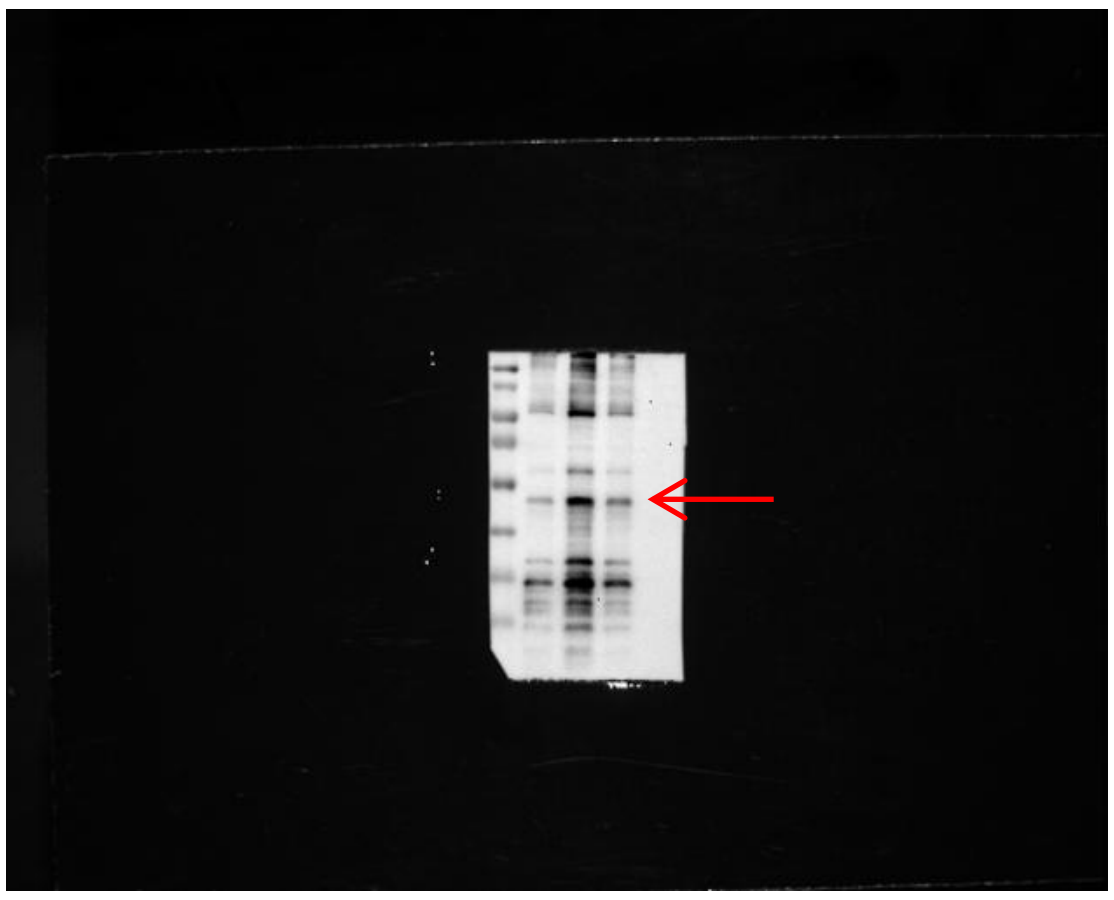
Tau	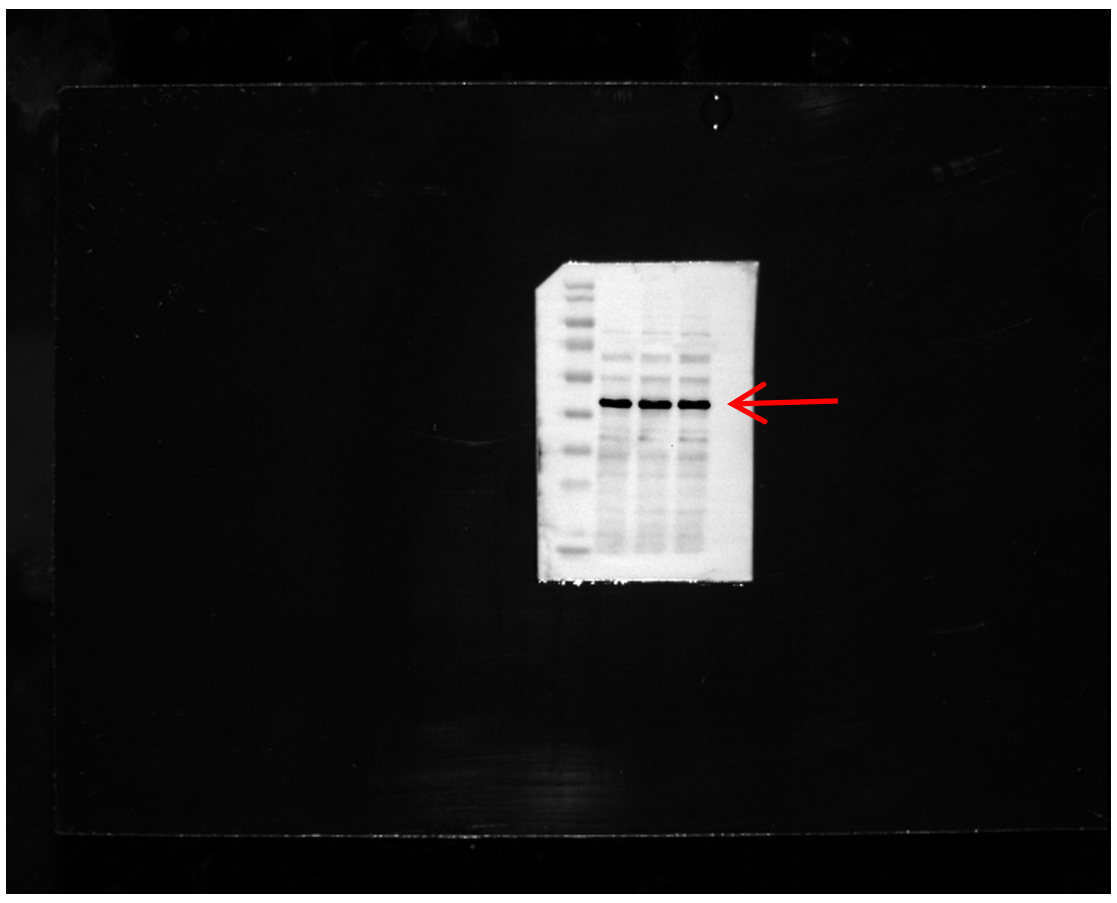
P-APP	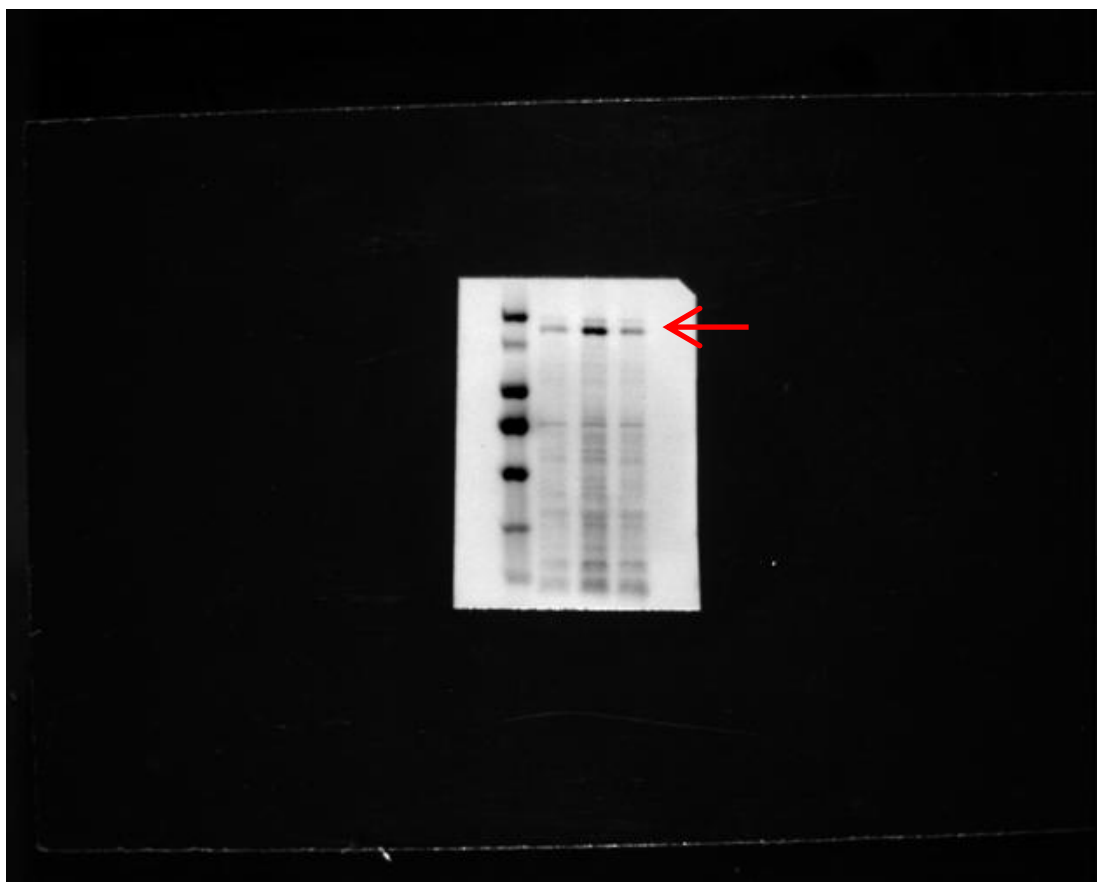
APP	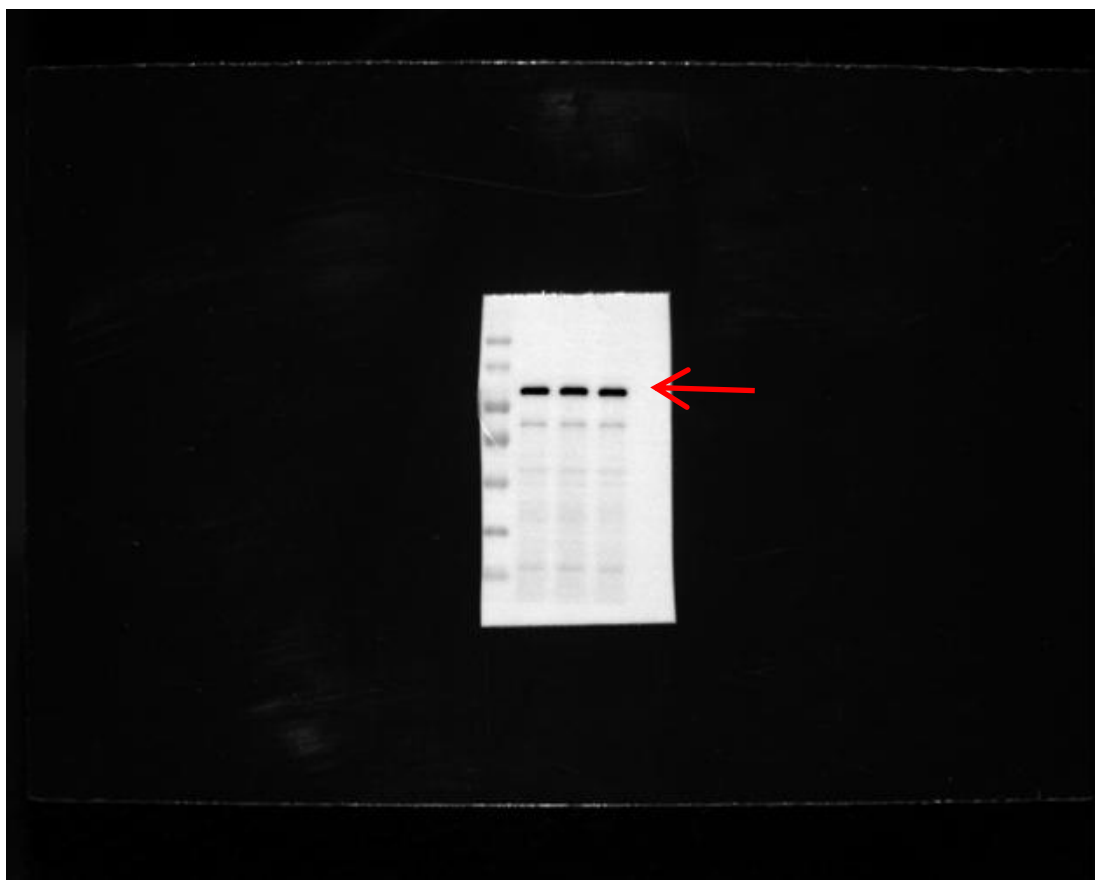

β-actin	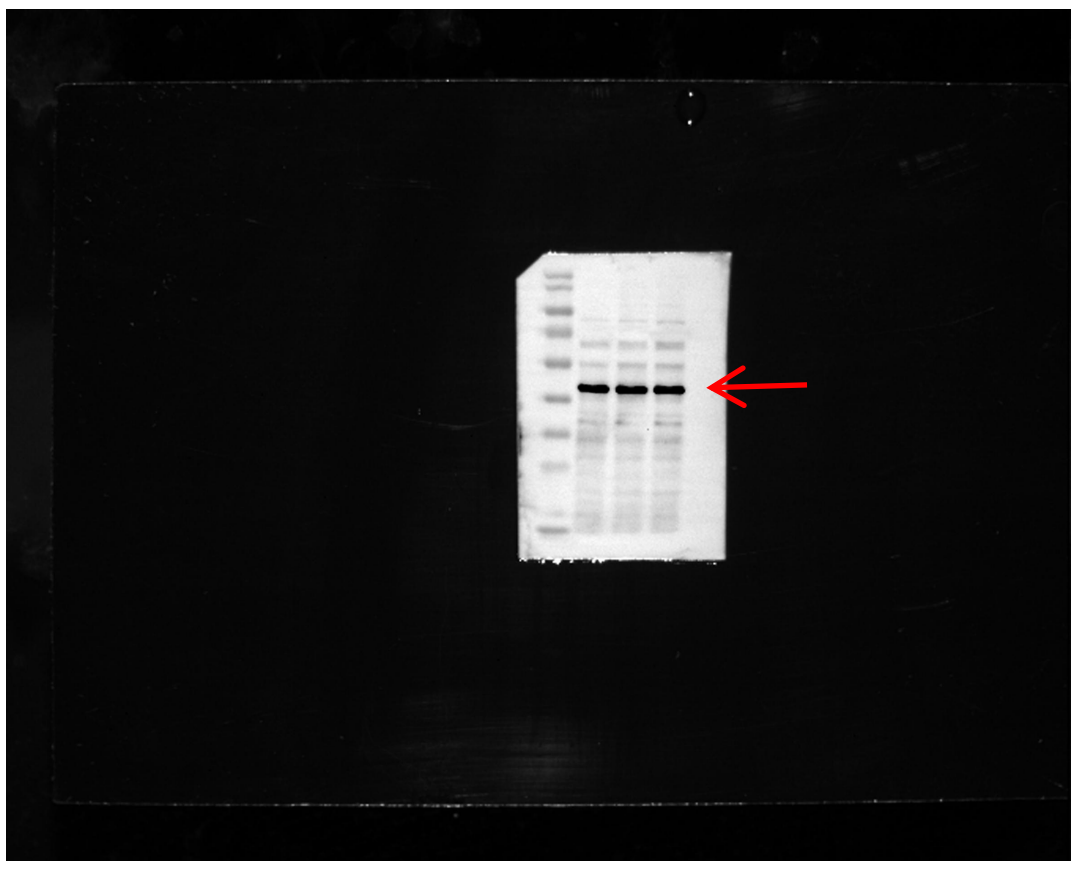

Figure 6

**SIRT3**	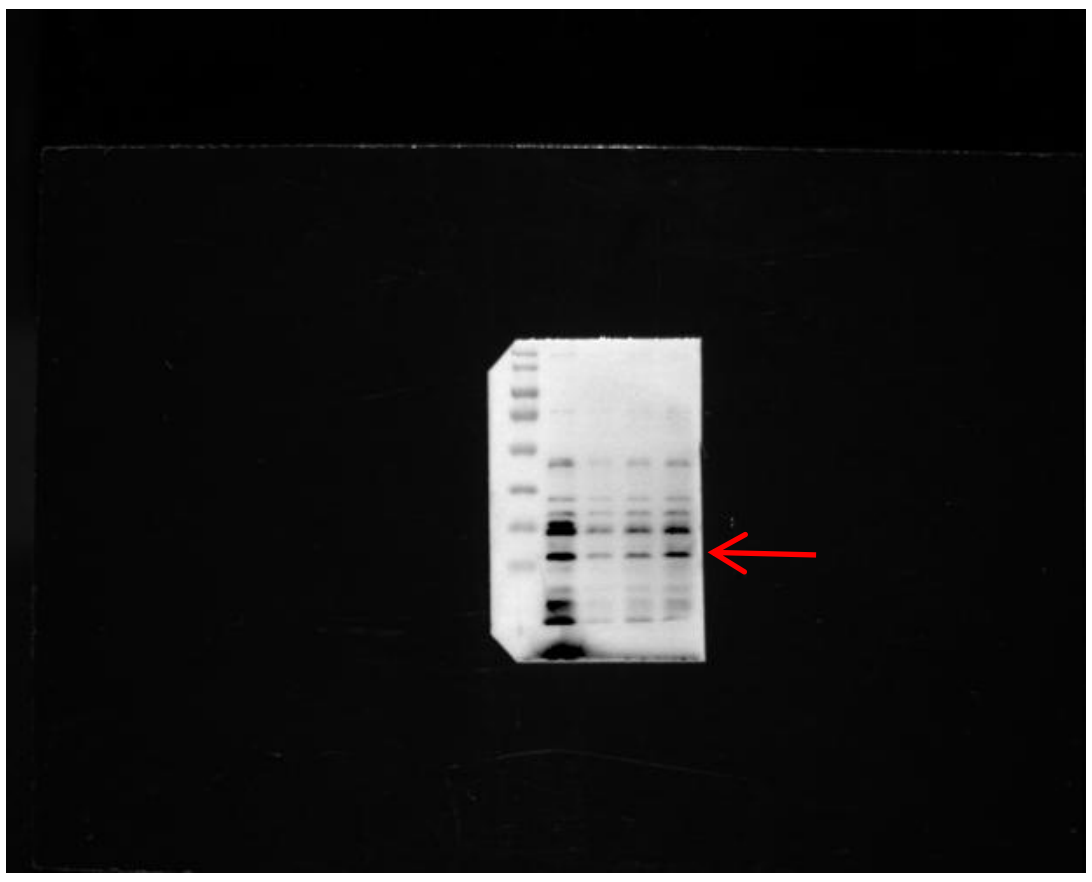
**β-actin**	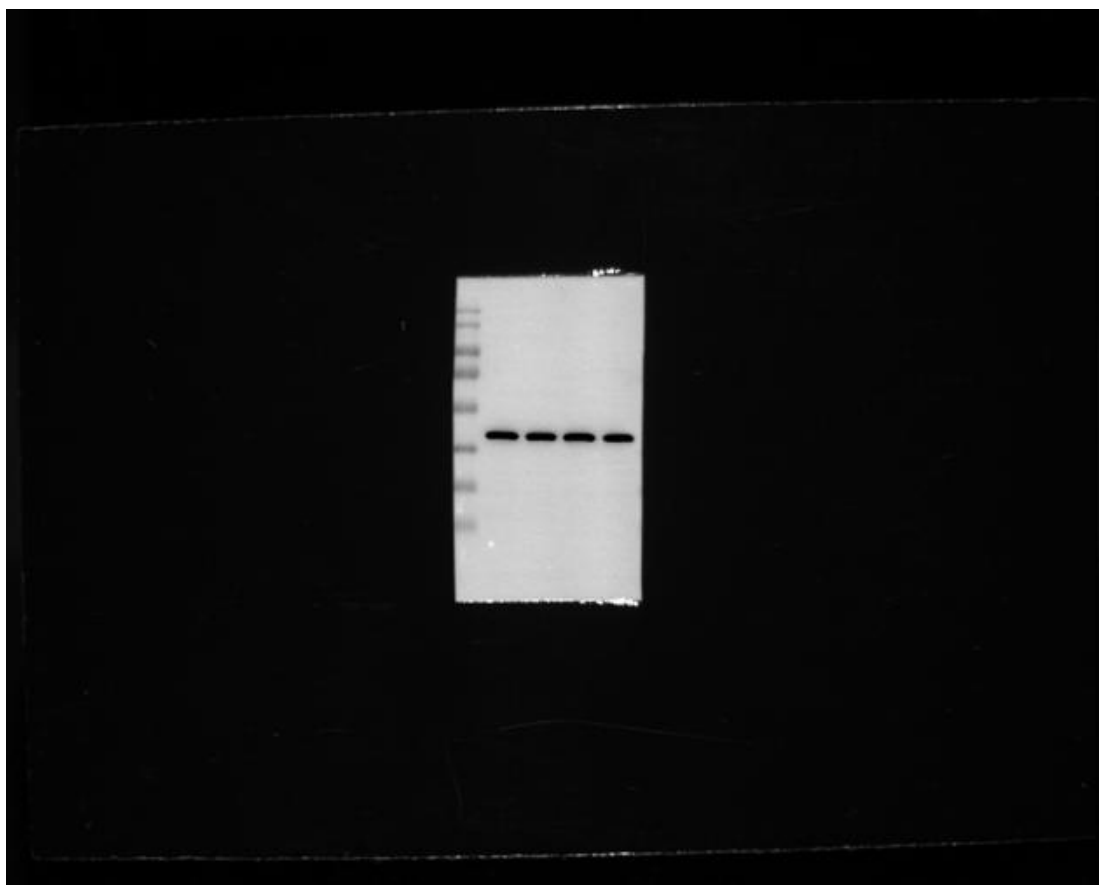

Figure 8

P-AMPK	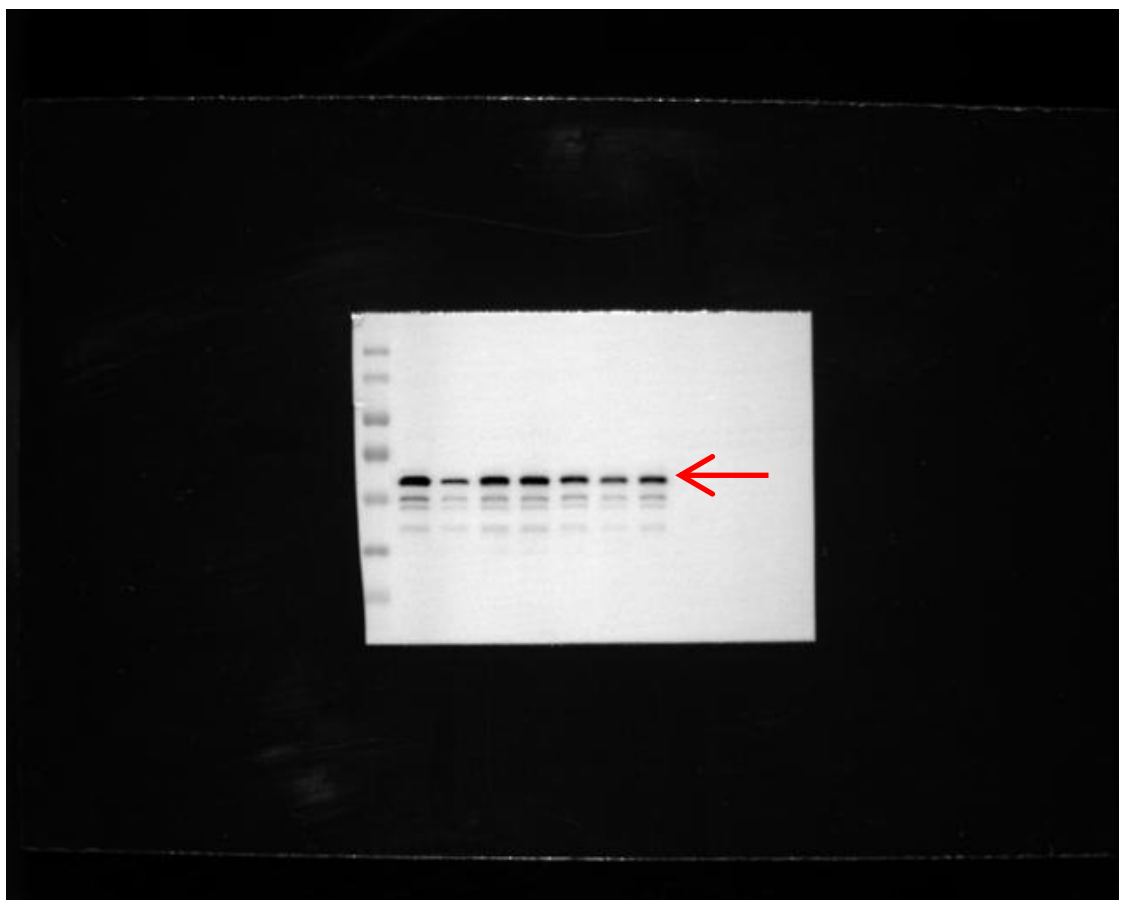
AMPK	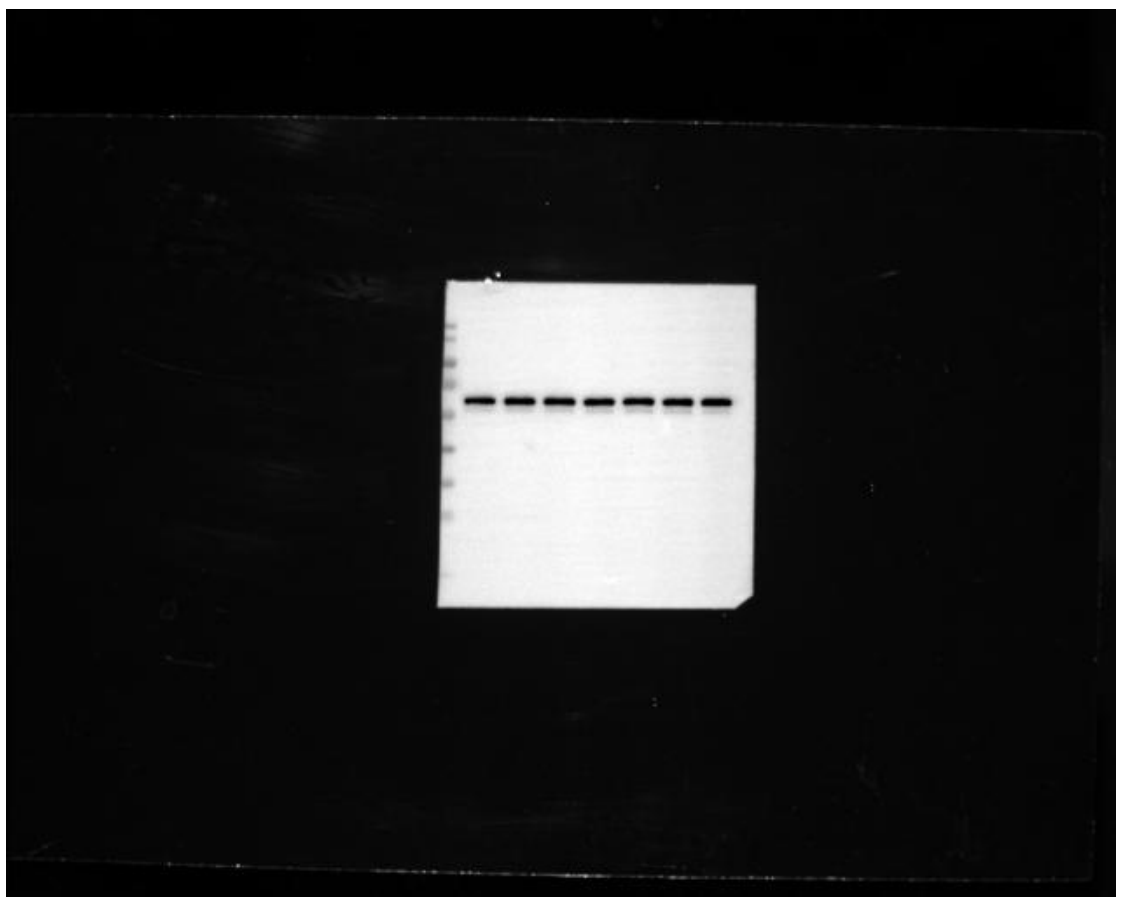
PGC-1α	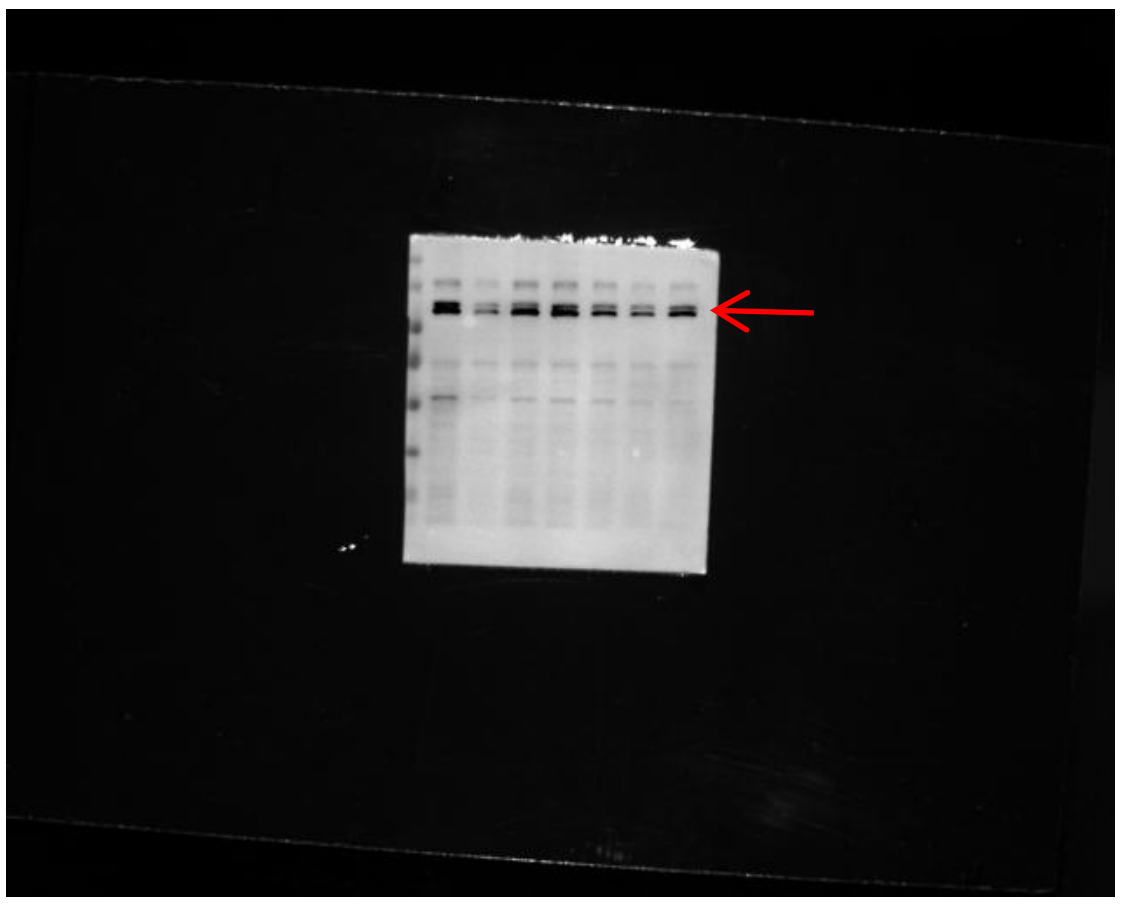
SIRT3	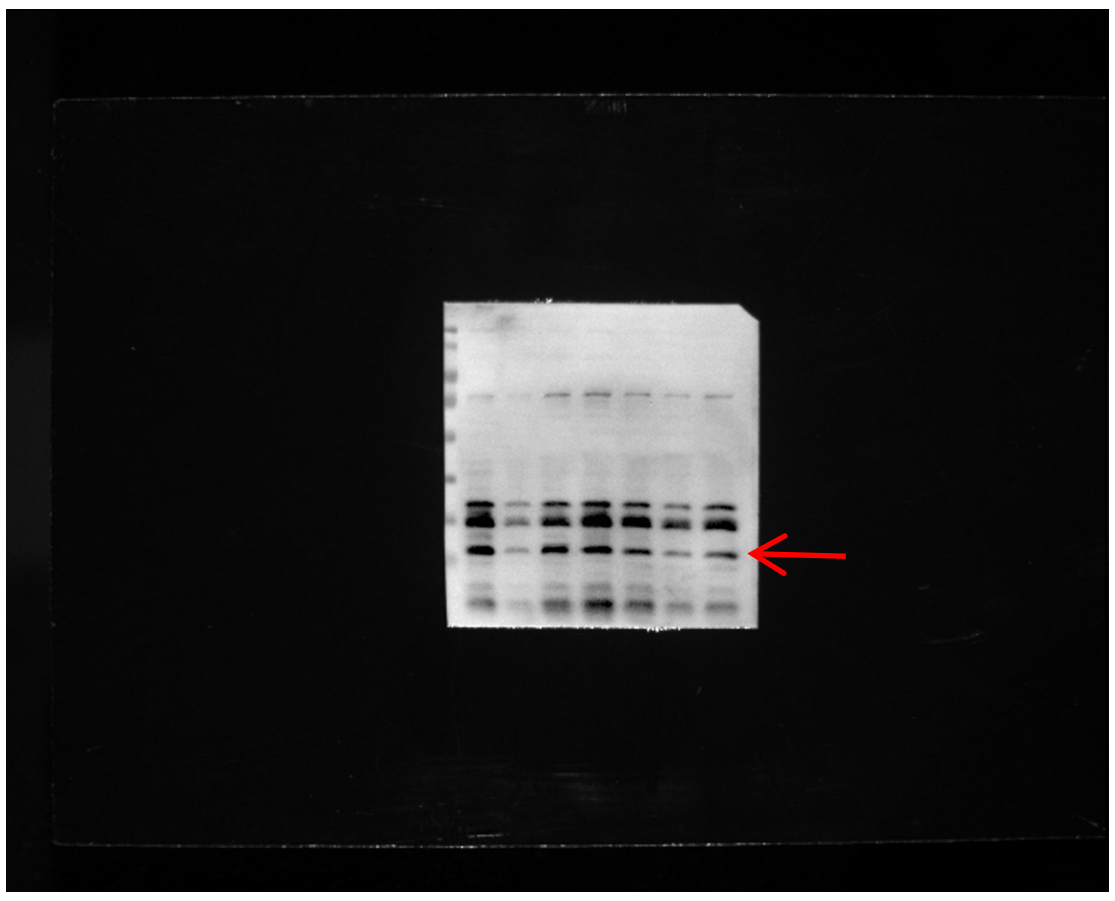

β-actin	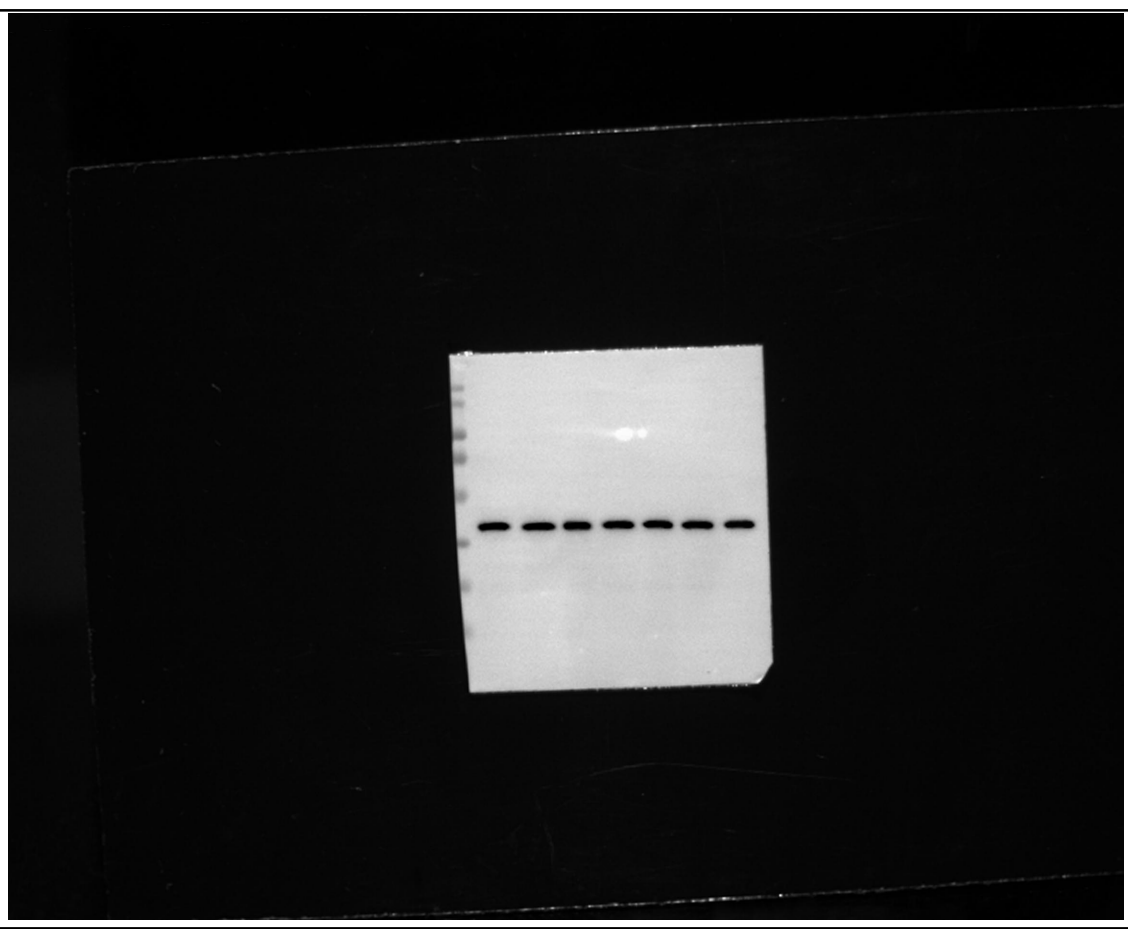
